# ACL injury management: a comprehensive review of novel biotherapeutics

**DOI:** 10.3389/fbioe.2024.1455225

**Published:** 2024-11-22

**Authors:** Xuezhi Yu, Jiahui Hu, Yifan Li, Yu Wen, Bin Li

**Affiliations:** ^1^ Department of Joint Surgery and Sports Medicine, Shengjing Hospital, China Medical University, Shenyang, China; ^2^ Department of Histology and Embryology, College of Basic Medical Sciences, China Medical University, Shenyang, China

**Keywords:** ACL injury, hyaluronic acid, self-assembled peptides, growth factors, stem cells, gene therapy, platelet-rich plasma, endogenous repair

## Abstract

The anterior cruciate ligament (ACL) is integral to the stability of the knee joint, serving to limit anterior tibial translation and regulate rotational movements. ACL injuries are among the most common and debilitating forms of knee trauma, often resulting in joint effusion, muscular atrophy, and diminished athletic capabilities. Despite the established efficacy of ACL reconstruction as the standard treatment, it is not uniformly successful. Consequently, there is a growing interest in novel biotherapeutic interventions as potential alternatives. This comprehensive review examines the latest advancements in ACL biotherapy, encompassing the application of hyaluronic acid, self-assembled short peptides, growth factors, stem cell therapy, gene therapy, platelet-rich plasma therapy, bone marrow aspirate concentrate cells, extracorporeal shock wave, electrical stimulation and cross bracing protocol. The collective aim of these innovative treatments is to facilitate the restoration of the ACL’s native biological and biomechanical integrity, with the ultimate goal of enhancing clinical outcomes and the functional recovery of affected individuals.

## 1 Introduction

Anterior cruciate ligament (ACL) injuries are a common orthopedic trauma that often alters athletic careers, posing a significant challenge to athletes and sports enthusiasts worldwide. These injuries affect over 200,000 individuals annually in the United States and have an incidence rate of 1/3,000 in Europe, resulting in costs exceeding $7 billion per year (Amini et al., 2023). The economic impact of ACL injuries is substantial, encompassing direct healthcare expenditures, rehabilitation costs, and indirect losses due to absenteeism and decreased productivity (Filbay et al., 2022). In a study assessing the injury burden and associated economic costs among professional European male football players (Pulici et al., 2022), results indicated an ACL injury incidence rate of 0.064 per 1,000 h of football match play, an injury burden of 14.4 days per 1,000 h, an average absence of 211.3 days due to ACL injuries, and the economic burden of ACL injuries was estimated at €84,499 per 1,000 h. Despite ongoing advancements in surgical techniques and rehabilitation protocols, a significant proportion of patients continue to experience persistent symptoms and functional impairments following ACL reconstruction, underscoring the necessity for innovative and effective treatment modalities (McDermott et al., 2023).

Surgical reconstruction (Amini et al., 2023; Yang et al., 2022), predominantly employing autografts or allografts, is the conventional gold standard for ACL injury management, with the objective of restoring knee stability and facilitating a return to pre-injury activity levels (Buerba et al., 2021). However, this approach is not unencumbered by limitations (Stockton et al., 2021; Benos et al., 2020). Postoperative complications, including graft failure, infection, and arthrofibrosis, can impede optimal outcomes (Zou et al., 2023; Tayfur et al., 2021; Zhao et al., 2023). Furthermore, the graft tissue utilized often lacks the intrinsic biological and biomechanical attributes of the native ACL, which may contribute to long-term joint degeneration (Leite et al., 2024). The reasons are as follows (Bauer et al., 2024; Johns, Vadhera, and Hammoud, 2024; Zhang et al., 2022; Gill et al., 2023; Knights et al., 2023; Park et al., 2022; Ramos-Mucci et al., 2022; Wang et al., 2018).

### 1.1 Complex tissue architecture

1) Multi-bundle Fiber Arrangement: The uniqueness of the ACL lies in its composition of multiple fiber bundles oriented in various directions, which play a crucial role at different stages of knee joint motion. Grafts typically consist of a single bundle or a few bundles, making it difficult to replicate this complex multi-bundle structure, thereby affecting their ability to provide stability.
2) Tissue Stratification: Collagen fibers within the ACL are not only orderly arranged but also exhibit stratified structures, which help maintain their function under variable stress conditions. Grafts often lack this intricate stratification, limiting their mechanical adaptability in different directions.

### 1.2 Blood supply and innervation

1) Abundant Blood Supply: The blood supply to the ACL is essential for its healing and metabolism. Grafts, due to the lack of rapid integration with the host’s vasculature, have a slow healing process, which may affect long-term stability and function.
2) Neurological Innervation: Sensory nerve endings within the ACL are crucial for the protective mechanisms of the knee joint. Grafts lack such innervation, potentially diminishing the sensory feedback and protective capabilities of the knee joint.

### 1.3 Biomechanical properties

1) Stress-Strain Characteristics: The ACL exhibits a unique stress-strain behavior, maintaining stability and elasticity under a wide range of stress conditions. Grafts often fail to replicate this characteristic, making them more susceptible to damage under high loads.
2) Biomechanical Adaptability: The ACL can adapt to the mechanical environment within the knee joint, maintaining joint stability. Grafts, in contrast, may lack this adaptability, potentially increasing the risk of joint degeneration over time.

### 1.4 Regenerative and repair capabilities

1) Cellular Composition: Fibroblasts and other cell types within the ACL are essential for its function and repair of injuries. The cellular composition of grafts may not fully replicate the cellular diversity and functionality of the native ACL.
2) Matrix Composition: The extracellular matrix (ECM) of the ACL, including collagen and proteoglycans, is crucial for supporting the ligament’s biomechanical functions and cellular activities. Differences in ECM composition of grafts may affect their long-term functional performance.

### 1.5 Immunogenicity

1) Allografts or artificial grafts may elicit an immune response, which can disrupt the healing process and lead to decreased graft function. Autologous ACL tissue, being from the same individual, rarely incites an immune rejection.

In light of these challenges, the research community has increasingly directed efforts towards the development of biological therapies designed to augment the healing and regeneration of the ACL, either as standalone interventions or in concert with surgical reconstruction (Murray, 2021).

Biological therapies for ACL injuries encompass a diverse array of strategies, such as the application of hyaluronic acid (HA), stem cells, growth factors, gene therapy, self-assembled short peptide (SAP), platelet-rich plasma (PRP), bone marrow aspirate concentrate cells (BMAC), extracorporeal shock wave (ESWT), electrical stimulation, cross bracing protocol (CBP) and various biomaterials applications. HA treatment for ACL injuries is a non-surgical approach primarily involving the injection of HA to alleviate knee joint pain, enhance joint function, and promote the natural healing process. The viscoelastic properties of HA contribute to the protection of articular cartilage, reduction of inflammatory responses, and creation of a more favorable environment for the repair of the injured ACL. Stem cell therapy for ACL injuries is a regenerative medicine approach that involves injecting stem cells into the site of injury to promote the natural repair and regeneration of the ligament. These cells possess the ability to differentiate into various cell types, including osteoblasts and chondrocytes, which contribute to enhancing the healing of the ACL and improving knee joint function. Growth factor therapy for ACL injuries primarily involves the local application of growth factors to facilitate ligament healing and tissue repair. These bioactive proteins stimulate cell proliferation, differentiation, and the synthesis of the extracellular matrix, thereby accelerating the recovery process of the injured ACL. Gene therapy for ACL injuries involves the introduction of specific genes into the injured ACL area to enhance the ligament’s self-repair capabilities. This approach utilizes genetic engineering techniques to promote the expression of growth factors and other beneficial proteins, thereby accelerating the healing process and improving the quality and function of the repaired tissue. Self-assembled short peptide treatment for ACL injuries involves the use of bioactive peptide sequences that can self-assemble into larger structures, providing a supportive scaffold for ligament repair. These peptides can stimulate cell migration, proliferation, and extracellular matrix production, thereby promoting the healing and regeneration of the damaged ACL. BMAC treatment for ACL injuries involves the use of concentrated autologous bone marrow cells, which are rich in growth factors and stem cells, to enhance the body’s natural healing response. This therapy leverages the regenerative potential of these cells to promote tissue repair, reduce inflammation, and improve the overall function of the injured ACL. ESWT for ACL injuries is a non-invasive treatment that utilizes high-energy sound waves to stimulate tissue healing and reduce pain. This method can enhance blood circulation, activate the body’s healing processes, and potentially improve the overall recovery of the injured ACL without the need for surgery. Electrical stimulation treatment for ACL injuries involves the application of low-voltage electrical currents to the affected area to enhance tissue healing and muscle function. This non-invasive therapy can stimulate nerve and muscle activity, reduce inflammation, and promote the regeneration of damaged ligament tissue, thereby supporting the recovery process after ACL injury. CBP for ACL injuries is a non-operative treatment approach that employs a specific knee brace to reduce strain on the ACL and support knee stability. This method aims to facilitate the healing process by limiting knee motion and providing a controlled environment for the injured ligament to recover, potentially reducing the need for surgical intervention.

**FIGURE 1:**
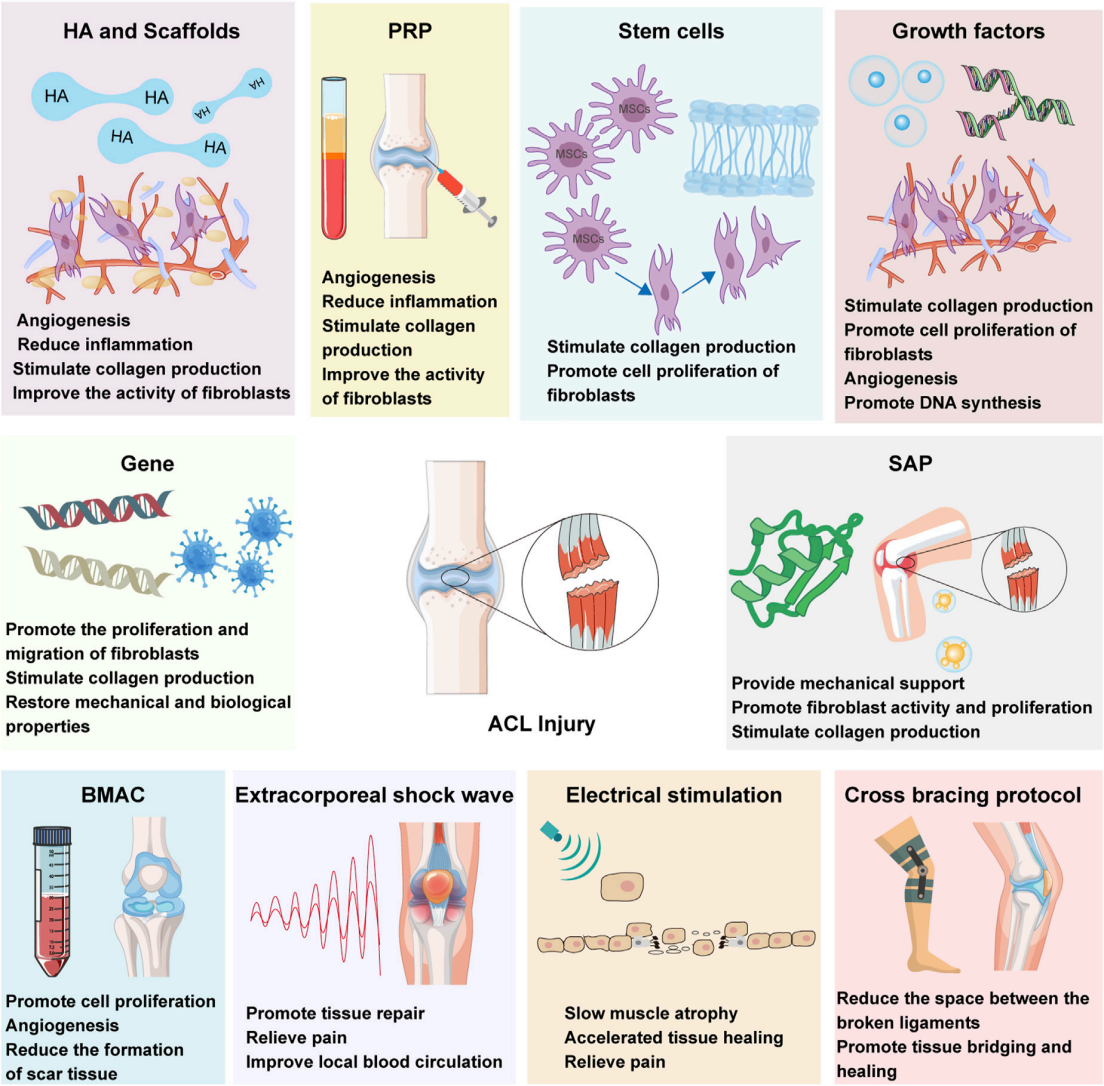
ACL biotherapeutics: hyaluronic acid (HA), stem cells therapy, growth factors, gene therapy, self-assembled short peptide (SAP), platelet-rich plasma (PRP) therapy, bone marrow aspirate concentrate cells (BMAC), extracorporeal shock wave (ESWT), electrical stimulation and cross bracing protocol (CBP).

These therapeutic modalities target the intricate biological processes inherent in ligament healing, including inflammation modulation, cellular recruitment and proliferation, ECM synthesis, and angiogenesis (Hassebrock et al., 2020; Chen et al., 2023; Xu et al., 2021). By fostering tissue regeneration and facilitating the restoration of the native ACL’s structural and functional integrity, biological therapies hold promise for enhancing clinical outcomes, mitigating the risk of post-traumatic osteoarthritis (PTOA), and ultimately, elevating the quality of life for individuals afflicted with ACL injuries (Figure 1).

This comprehensive review endeavors to provide an exhaustive overview of the current landscape of biological therapies for ACL injury management. By meticulously reviewing the current body of literature, we will assess the evidence supporting the efficacy, safety, and feasibility of these therapeutic approaches, discuss their potential benefits and drawbacks, and outline promising directions for future research. The goal of this review is to inform clinical decision-making, steer subsequent research endeavors, and contribute to the formulation of more efficacious, personalized, and biologically informed strategies for the management of ACL injuries.

## 2 ACL structural characteristics

The ACL is strategically positioned within the knee joint’s central region, originating from the anterior medial margin of the tibial plateau’s intercondylar area. It courses in an arcuate trajectory posteriorly and medially, ultimately anchoring to the lateral condyle of the femur, thereby creating a distinct and nearly fan-shaped architectural arrangement. The ACL’s ingenious anatomical design not only augments its coverage area to provide comprehensive and stable support to the knee joint, but also ingeniously circumvents contact with the intercondylar fossa upon full knee extension, thereby minimizing the risk of friction-induced damage (Fink et al., 2018).

From an anatomical and functional perspective, the ACL is naturally bifurcated into two complementary functional units based on their tibial attachment sites: the anterolateral (AL) and posterolateral (PL) bundles. The AL bundle is situated in the posterior superior aspect of the femur and the anterolateral region of the tibia, with its tension peaking during knee flexion. Its primary function is to effectively curtail excessive tibial translation along the anterolateral axis. Conversely, the PL bundle is located in the anterior inferior femur and the posterior inferior tibia. Upon knee extension, its tension markedly increases, serving to prevent improper tibial rotation.

The ACL’s microstructure is characterized by a highly organized and compact connective tissue matrix, replete with a dense array of collagen fibers, a modest amount of interstitial fibers, and fibroblasts with distinctive morphological features. The collagen fiber bundles constitute the fundamental framework of the ACL’s mechanical robustness, endowing it with the capacity to withstand tensile and torsional forces. These fibers are not discrete entities but are ensheathed in a matrix, predominantly composed of proteoglycans, which impart the ligament with essential viscoelastic properties and hydration capabilities (Markatos et al., 2013; Hoffmann and Gross, 2007).

Under normal physiological conditions, the ACL maintains a high-water content, ranging from 60% to 70%, reflecting its optimal hydration characteristics. Upon desiccation, approximately 75% of its dry weight is attributable to collagen fibers of types I, III, IV, and VI, which are meticulously arranged and interwoven to confer the ACL with its superior mechanical attributes. The remaining 25% of the dry weight is composed of fibroblasts, elastin, and other bioactive substances, including proteoglycans. Notably, despite elastin’s minor proportion of the total weight (approximately 1%), it plays an indispensable role in maintaining the ACL’s elastic properties, enabling the ligament to swiftly revert to its original configuration post-loading and ensuring the smooth and efficient functioning of the knee joint (Markatos et al., 2013; Hoffmann and Gross, 2007).

ACL is situated at the center of the knee joint, originating from the anterior medial margin of the intercondylar area of the tibial plateau. It courses in a posterior and medial direction, ultimately anchoring to the lateral condyle of the femur, creating a unique and nearly fan-shaped architectural arrangement. The ingenious anatomical design of the ACL not only increases its coverage area, providing comprehensive and stable support to the knee joint, but also cleverly avoids contact with the intercondylar fossa upon full knee extension, thereby minimizing the risk of friction-induced damage (Fink et al., 2018). From an anatomical and functional perspective, the ACL is naturally bifurcated into two complementary functional units, the anterolateral (AL) and posterolateral (PL) bundles, depending on their tibial attachment sites. The AL bundle, located on the posterior superior aspect of the femur and the anterolateral region of the tibia, reaches peak tension during knee flexion. Its primary function is to effectively curb excessive tibial translation along the anterolateral axis. In contrast, the PL bundle, located on the anterior inferior femur and the posterior inferior tibia, experiences a significant increase in tension upon knee extension, serving to prevent improper tibial rotation.

**FIGURE 2:**
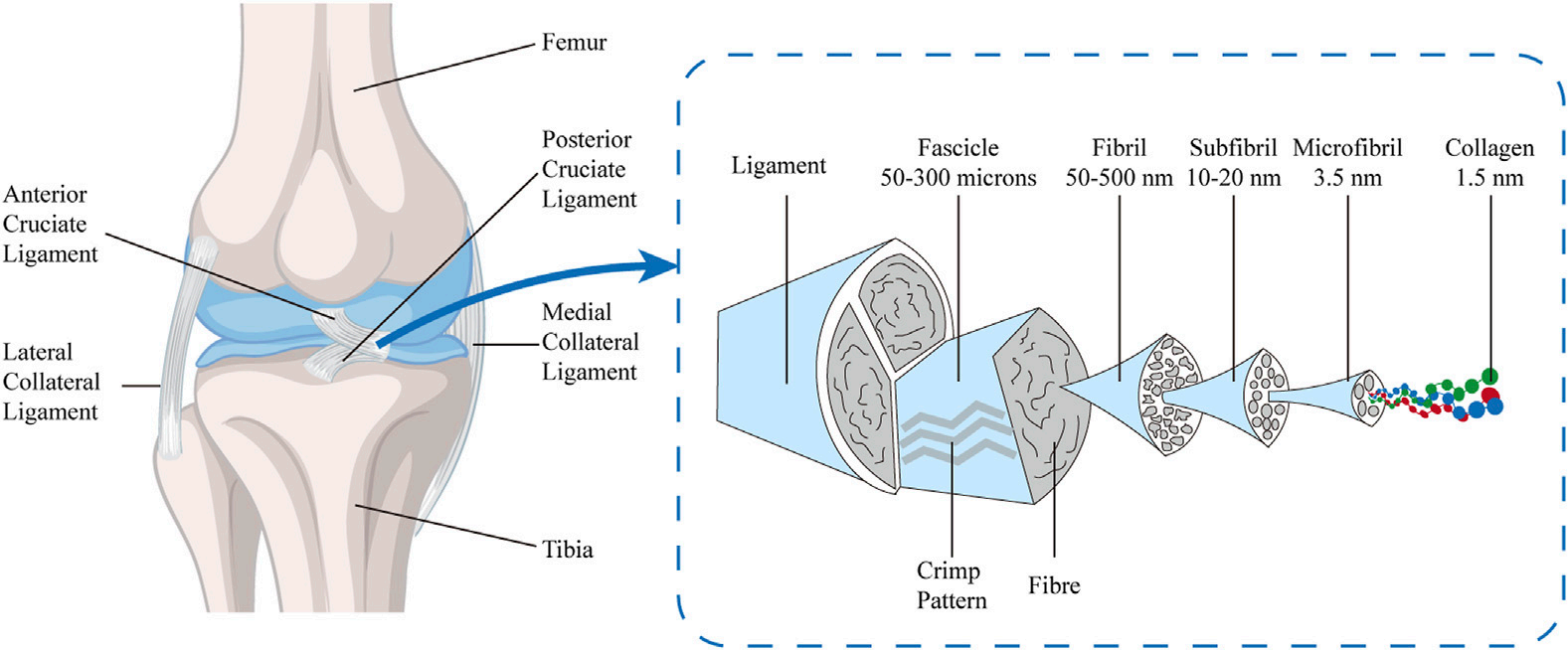
Schematic drawing of the multi-unit hierarchical structure of ACL.

The microstructure of the ACL is characterized by a highly organized and compact connective tissue matrix, rich in collagen fibers, a moderate amount of interstitial fibers, and fibroblasts with distinctive morphological features. The collagen fiber bundles form the fundamental framework of the ACL’s mechanical robustness, endowing it with the capacity to withstand tensile and torsional forces. These fibers are not discrete entities but are ensheathed in a matrix, predominantly composed of proteoglycans, which confer essential viscoelastic properties and hydration capabilities to the ligament (Markatos et al., 2013; Hoffmann and Gross, 2007). Elastic fibers, matrix proteins (such as proteoglycans), and other components together constitute the composite structure of the ACL. The interactions between these components enhance the ligament’s toughness and elasticity, further improving its functional performance in complex movements. The structural design of the ACL is not only for resisting external forces but also for adaptively adjusting to the mechanical loads experienced by the knee joint. Its anisotropic structure provides varying mechanical support at different angles and activities of the knee, maintaining joint stability. The complex interplay of these structural components and their orientation is crucial for the biomechanical function of the ACL and is an important consideration in the study and treatment of ACL injuries. Under normal physiological conditions, the ACL maintains a high-water content, ranging from 60% to 70%, reflecting its optimal hydration characteristics. Upon desiccation, approximately 75% of its dry weight is attributable to collagen fibers of types I, III, IV, and VI, which are meticulously arranged and interwoven to confer superior mechanical properties on the ACL. The remaining 25% of the dry weight consists of fibroblasts, elastin, and other bioactive substances, including proteoglycans. Notably, despite elastin’s minor proportion of the total weight (about 1%), it plays an indispensable role in maintaining the elastic properties of the ACL, enabling the ligament to quickly revert to its original configuration after loading and ensuring the smooth and efficient functioning of the knee joint (Markatos et al., 2013; Hoffmann and Gross, 2007). The ACL possesses a hierarchically and sequentially assembled tissue, with increasing diameter and mechanical strength, including collagen molecules (triple-helical polypeptide chains, diameter < 2 nm), which form microfibrils (diameter 3.5 nm) through cross-linking. These microfibrils arrange themselves into subfibrils (diameter 10–20 nm), which then form into fibrillar bundles (50–300 μm) within the fibrils (diameter 50–500 nm), repeating a crimped pattern every 45–60 μm. They are cross-linked to form subunits parallel to the long axis of the tissue, also containing proteoglycans and elastin, and are surrounded by a vascularized sheath, forming the ligament (Duthon et al., 2006; Connizzo, Yannascoli, and Soslowsky, 2013; Dourte, Kuntz, and Soslowsky, 2008; Gomez-Rodriguez et al., 2007) (Figure 2).

The ACL exhibits anisotropy, with collagen fibers within the ligament demonstrating different physical properties in various directions. Most collagen fibers are aligned along the primary axis of the ligament to enhance strength and stiffness in the direction of the principal stress. This highly directional arrangement endows the ACL with uniaxial anisotropy, meaning it has the highest tensile strength and stiffness along its main axis, which is crucial for resisting the anterior translation of the tibia relative to the femur and maintaining knee joint stability. The stress-strain response of the ACL, due to the oriented arrangement of its collagen fibers, exhibits a nonlinear relationship. This allows the ACL to display greater flexibility at low stress levels, with increasing stiffness as stress rises. This nonlinear response helps the ACL maintain stability across the range of knee joint motion. Furthermore, the anisotropic structure of the ACL provides excellent fatigue resistance under repetitive loading, as the collagen fibers are aligned along the main stress direction, enabling the ACL to maintain its structural integrity during long-term dynamic activities (Pardo et al., 2024).

## 3 ACL biomechanics

The ultimate tensile strength of the ACL is estimated to be within the range of approximately 1725 ± 270 N. The stress-strain relationship of the ACL can be delineated into three distinct phases: the toe region, the linear region, and the yield region. In the initial toe phase, characterized by lower stress levels, the ACL’s collagen fibers, which are initially coiled, progressively extend. As the stress escalates and enters the linear phase, the fibers cease to elongate, yet the resistance they offer incrementally rises. Upon reaching the yield phase, the collagen fibers experience permanent deformation and eventual rupture, culminating in a reduction of stress that can precipitate ligamentous failure (Noyes et al., 1984).

During the static stabilization of the knee, the ACL assumes a pivotal role in impeding the anterior translation of the femur in relation to the tibia, accounting for roughly 86% of the resistive forces exerted. Throughout various phases of knee joint motion, distinct components of the ACL contribute differentially to the joint’s stabilization. Specifically, the anteromedial bundle tightens at approximately 90° of knee flexion, while the posterolateral bundle becomes taut as the knee approaches full extension. It is noteworthy that the ACL exerts a relatively minimal influence in resisting rotational movements within the knee joint, such as internal and external rotations (Lohmander et al., 2004; Datta et al., 2019).

## 4 ACL endogenous repair

Following an injury to the ACL, the capacity for endogenous healing is markedly constrained, resulting in a protracted reparative process. The scar tissue that forms after healing is incapable of fully recapitulating the intricate biological and mechanical attributes of the original ACL tissue (Zou et al., 2023; Yang et al., 2022). This limitation in the ACL’s self-restorative potential is a significant factor contributing to the persistence of functional deficits and the predisposition towards the development of post-traumatic osteoarthritis in the affected knee joint.

### 4.1 ACL endogenous repair phases

The repaired tissue post-injury invariably fails to autonomously replicate the intricate biological and mechanical properties of the original ACL tissue (Brinlee et al., 2022). The endogenous healing cascade of the ACL is typically characterized by three distinct yet interconnected phases:

1) The inflammatory phase, spanning from the onset of injury up to approximately 8 weeks, is marked by localized plasma exudation, fibrin clot formation, and the infiltration of monocytes, leukocytes, and macrophages. Concurrently, there is a release of pro-angiogenic and proliferative cytokines that initiate the healing response.
2) The proliferative phase commences around the 8-week post-injury mark, during which neovascularization occurs, fibroblasts proliferate, and the nascent collagen matrix begins to infiltrate and fill the compromised regions of the ligament.
3) The remodeling phase, which typically unfolds between 1 and 2 years post-injury, is defined by a reduction in local cellularity and a reorganization of the extracellular matrix under the influence of mechanical stressors (Murray et al., 2000; Woo, Vogrin, and Abramowitch, 2000; Murray and Fleming, 2013).

While these stages constitute the cornerstone of the ACL’s natural reparative mechanisms, the inherent limitations of this healing process often necessitate the pursuit of supplementary interventions. Such treatments may include ACL reconstructive surgery or alternative biological therapies, which are employed with the aim of restoring knee stability and functionality. These interventions are critical in addressing the shortfalls of the natural healing process and facilitating a more comprehensive and durable restoration of the ACL’s structural and functional integrity.

### 4.2 Factors affecting ACL endogenous repair

The suboptimal endogenous healing response of the ACL is significantly influenced by intra-articular factors such as synovial fluid and mechanical motion. These elements can compromise the stability of the fibrin-platelet scaffold and the integrity of blood clot formation, both of which are crucial for the initial stages of ACL injury repair and the subsequent reparative processes (Murray et al., 2007). At the molecular level, the synovial fluid in patients with ACL injuries exhibits elevated levels of matrix metalloproteinase 3 (MMP-3), interleukin 6 (IL-6), and tissue inhibitor of metalloproteinases 1 (TIMP-1) (Higuchi et al., 2006). MMPs and TIMPs play pivotal roles in the tissue remodeling process, and an imbalanced MMP/TIMP ratio can predispose knee joints to cartilage degradation. Furthermore, an accumulation of MMPs within the joint can hinder the ACL’s healing process (Higuchi et al., 2006; Hirzinger et al., 2014). Higuchi et al. have reported that post-ACL injury, MMP-3 concentrations remain elevated irrespective of the time elapsed since injury, while TIMP-1 levels decrease, implying that the timing of therapeutic intervention may be a critical factor in the management of ACL injuries (Higuchi et al., 2006).

Additionally, the scarcity of repair-related cytokines (growth factors) and wound-filling compounds such as fibrinogen and fibronectin can impede the healing process. The absence of a continuous collagen fiber network between the old and newly formed extracellular matrices may also attenuate the mechanical resilience of the ACL tissue (Murray and Fleming, 2013; Murray et al., 2000; Murray, 2009). This disruption in collagen continuity can lead to a diminished capacity to withstand physiological stresses, further complicating the restoration of the ACL’s structural and functional integrity.

Research indicates that dedifferentiated bone marrow mesenchymal stem cells (De-BMSCs) significantly enhance the early biomechanical strength of anterior cruciate ligament reconstruction (ACLR) by activating the Nanog/NFATc1/Osterix signaling pathway and promote tendon-bone healing. These De-BMSCs possess the capability to differentiate into a variety of tissue cells, including chondrocytes, osteoblasts, and epithelial cells, which contribute to the reconstruction of the complex transitional structure at the tendon-bone interface (Tie et al., 2021). Macrophages polarize into M1 or M2 types based on the signals they receive, with M1 type predominating in the early stages of healing and secreting pro-inflammatory cytokines, while M2 type exhibits anti-inflammatory and reparative functions, accumulating at the site of injury over time. Mesenchymal stem cells (MSCs) respond to the inflammatory environment through their immunomodulatory properties, secreting anti-inflammatory factors such as prostaglandin E2 (PGE2), and promoting the shift of macrophages towards the M2 anti-inflammatory phenotype. Additionally, factors secreted by MSCs and macrophages, such as vascular endothelial growth factor (VEGF) and basic fibroblast growth factor (bFGF), stimulate neovascularization, providing essential nutrients and oxygen to the healing area. The secretion of these factors is crucial for the healing of the tendon-bone interface after ACL reconstruction, as they participate in the synthesis and remodeling of the extracellular matrix, involving various growth factors and cytokines, such as platelet-derived growth factor (PDGF), transforming growth factor-β (TGF-β), and bone morphogenetic proteins (BMPs). MSCs can also regulate the behavior of macrophages and promote their anti-inflammatory and tissue repair functions by releasing exosomes containing miRNA and other bioactive molecules. Studies by Tie et al. (2021), Xu et al. (2021) emphasize the importance of these cellular interactions in regulating inflammatory responses and promoting tissue healing. Research by Amini et al. (2023) demonstrates that the overexpression of TGF-β and FGF-2 in human ACL fibroblasts not only promotes the deposition of extracellular matrix components such as type I/III collagen, proteoglycans/decorin, and Tenascin-C but also increases the expression levels of specific transcription factors like Mohawk and scleraxis, which are crucial for the tendon-bone healing process. Concurrently, this overexpression process does not activate inflammatory mediators IL-1β and tumor necrosis factor-α (TNF-α).

## 5 Biological treatments

### 5.1 Hyaluronic acid (HA) and other scaffolds therapy

Hyaluronic acid (HA), a linear polysaccharide, is ubiquitously present within the extracellular matrix and synovial fluid of various connective tissues. HA exerts a significant influence on cellular processes, including proliferation and differentiation, through its modulation of cell-matrix interactions (Skandalis et al., 2020; Iaconisi et al., 2023; Knapik et al., 2021). These regulatory mechanisms underscore the pivotal role of HA in the realms of tissue repair and cellular biology. By facilitating dynamic interactions between cells and their surrounding matrix, HA contributes to the orchestration of cellular behaviors that are integral to the maintenance of tissue homeostasis and the restoration of tissue integrity following injury (Marinho et al., 2021; Di Mola et al., 2022).

#### 5.1.1 HA and animal models

Investigations into the therapeutic application of HA for the treatment of ACL injuries are predominantly in the preclinical phase. Dating back to 1990, a seminal study demonstrated that the administration of HA injections could foster ligamentous healing by augmenting angiogenesis and mitigating inflammatory responses during the early reparative phase of partial-ACL tears in a rabbit model (Wiig et al., 1990). Subsequent research by Yoneda et al. indicated that the intra-articular injection of high molecular weight HA into injured ACLs of rabbits not only enhanced collagen remodeling but also stimulated an increase in fibroblast activity, indicative of a heightened collagen synthesis. Concurrently, HA treatment led to a significant attenuation of inflammatory responses and a proliferation of fibroblasts within the regions of tissue repair. However, it is noteworthy that despite 12 weeks of treatment with high molecular weight HA, a non-healing rate of 33.33% was observed (Wiig et al., 1990). Furthermore, HA degradation products, specifically disaccharide fragments ranging from 4 to 25 units in length, have been shown to promote angiogenesis and provide a structural framework conducive to ACL repair (Yoneda et al., 1988a; Yoneda et al., 1988b). Collectively, these findings suggest that while HA holds promise as a therapeutic agent for ACL injury, further research and refinement of HA-based treatment modalities are imperative to optimize their clinical efficacy and safety. In a study of the effects of intra-articular injection of hyaluronic acid (HA) on the biomechanical properties and histological changes of the anterior cruciate ligament (ACL) in rats, the results showed that intra-articular injection of HA did not significantly affect the tensile strength of the ACL in rats, and no significant effects were observed on other markers of degenerative change (Yucel et al., 2009). In Balasubramaniyan’s study, researchers engineered an ultra-high molecular weight polyethylene (UHMWPE) braided structure for anterior cruciate ligament (ACL) reconstruction, enhancing its biocompatibility through innovative surface modifications. The UHMWPE was treated with low-temperature plasma and subsequently coated with cationized gelatin and hyaluronic acid, creating a construct that not only upheld the integrity of the ligament but also fostered a favorable environment for integration with native tissues. The *in-vitro* cytotoxicity and genotoxicity assays confirmed the non-toxic and non-mutagenic nature of the ACL extract, while *in-vivo* analyses revealed that the treated ligament was non-irritating, non-sensitizing, and actively promoted the formation of new tissue around the graft (Balasubramaniyan et al., 2022).

#### 5.1.2 HA and human ACL repair

In the evolutionary journey of tissue engineering, the year 2008 marked a significant milestone with the introduction of HA into human clinical trials, specifically for the purpose of reconstructing engineered ligament tissue. Pioneering work by Young-Kwon and colleagues led to the development of an innovative composite scaffolding system. This system integrated fiber materials within an HA matrix and was successfully transplanted *in vivo*, demonstrating not only excellent biocompatibility but also significant promotion of neovascularization and cell migration, as evidenced by Seo et al. (2009), Kwon et al. (2013). These findings have laid a critical theoretical and empirical groundwork for the use of HA as a pivotal component in the construction of tissue-engineered ligaments, particularly for the repair of damaged ACL. ACL and meniscus injuries, prevalent among athletes and the general population, can lead to early post-traumatic osteoarthritis in 50–60 percent of patients, regardless of whether reconstructive surgery is performed. The anti-inflammatory, anabolic, and chondroprotective effects of hyaluronic acid have been demonstrated *in vitro* and in animal models. Intra-articular injections of hyaluronic acid have proven beneficial in young patients with acute knee injuries, including symptomatic meniscal tears and isolated ACL injuries with cartilage damage. As noted by Jazrawi and Rosen (2011), intra-articular injections of hyaluronic acid are efficacious for non-traumatic osteoarthritis and are endorsed by current treatment guidelines (Jazrawi and Rosen, 2011). The seamless integration of HA into scaffold design for ligament tissue engineering signifies a notable advancement in the field, presenting a promising pathway for the development of more efficacious ACL repair strategies. A study conducted by Hu et al. (2023) aimed to evaluate the therapeutic benefits of combining arthroscopic anterior cruciate ligament reconstruction (AACLR) with sodium hyaluronate (SH) in ACL injury patients, both with and without early knee osteoarthritis (KOA). The inflammatory response was meticulously monitored using enzyme-linked immunosorbent assay (ELISA) for serum inflammatory markers. Knee functionality was comprehensively assessed using the lysholm knee score, the international knee documentation committee (IKDC) knee evaluation form, and by measuring the range of motion of the knee joint. The study concluded that AACLR combined with SH effectively improves knee function and reduces inflammation in ACLI patients, irrespective of their KOA status (Hu et al., 2023). Despite the progress of tissue engineering, which has given rise to numerous bioengineering techniques for the repair of ACL injuries, the majority of these techniques remain in the preclinical animal experiment stage. Translation to widespread clinical practice has been gradual, underlining the need for further research and development to harness the full potential of these advancements in orthopedic medicine.

#### 5.1.3 Other scaffolding materials

##### 5.1.3.1 Aligned gelatin microribbon scaffolds with hydroxyapatite gradient

This study introduces a pioneering strategy in the realm of tissue engineering, specifically tailored to surmount the reparative challenges encountered at the bone-tendon interface. The investigators have crafted an innovative aligned gelatin microribbon (μRB) hydrogel scaffold, replete with a meticulously designed gradient of hydroxyapatite nanoparticles (HA-np). This construct is meticulously engineered to orchestrate the zonal-specific differentiation of human mesenchymal stem cells (hMSCs), effectively emulating the intricate natural gradient of the bone-tendon interface. The aligned μRBs within the scaffold serve as a structural guide, promoting cell alignment in three-dimensional space. Moreover, the HA-np gradient is instrumental in driving the mesenchymal stem cells towards a differentiation pathway that closely mirrors the native tissues at the bone-tendon junction. The introduction of a brief chondrogenic priming phase, preceding exposure to osteogenic factors, refines this mimicry of the bone-cartilage-tendon transition, substantially augmenting the tensile modulus of the resulting engineered tissues. Divergent from prior methodologies, which often necessitated the use of composite materials and multilayer scaffolding techniques, this research presents a paradigm shift with a continuous scaffold system that capitalizes on a single cell source. The 3D macroporous microribbon hydrogel platform, featuring aligned structural cues in conjunction with the zonal integration of hydroxyapatite nanoparticles, facilitates seamless differentiation across the bone-tendon interface. This unified approach not only streamlines the tissue engineering process but also holds great promise for enhancing the functional and mechanical properties of the engineered interface, thereby advancing the field of regenerative medicine (Stanton et al., 2022).

##### 5.1.3.2 Bioactive strontium substituted hydroxyapatite for polyethylene terephthalate artificial ligaments

In this thorough investigation, the capacity of strontium-substituted hydroxyapatite (SrHA) nanoparticles to augment the bioactivity and osseointegration of polyethylene terephthalate (PET) artificial ligaments, extensively applied in ACL reconstruction surgeries, has been rigorously evaluated. The prevalent use of PET ligaments, while beneficial, is often compromised by the challenge of suboptimal healing at the ligament-bone interface. Through an innovative microwave-hydrothermal synthesis, SrHA nanoparticles with varying strontium content were developed and applied as a coating to PET ligaments, significantly modulating their chemical composition and crystallinity without disrupting the phase or morphology. The application of these nanoparticles was confirmed through scanning electron microscopy (SEM), which provided visual evidence of their successful and uniform deposition onto the PET ligament surfaces, concurrent with a marked enhancement in surface hydrophilicity due to the coatings. This surface modification is anticipated to improve the interaction with biological systems and promote osseointegration. *In vitro* studies illuminated the impact of Sr ion concentrations on the osteogenic potential of rat bone marrow mesenchymal stem cells (rBMSCs), with the 2SrHA-PET group evidencing the most favorable osteogenic differentiation. This was underscored by the upregulation of key osteogenic genes BMP-2, OCN, Col-I, and VEGF, which are integral to bone formation and repair processes. The *in vivo* studies corroborated these promising *in vitro* results, demonstrating that the 2SrHA-PET group possessed an enhanced osteogenic capacity, leading to superior ligament-bone integration and increased biomechanical strength when compared to groups treated with PET and HA-PET. The research culminates in the compelling conclusion that SrHA nanoparticle coatings offer a promising strategy to bolster osseointegration in ligament repair. This innovative application presents a transformative modification method for PET artificial ligaments in ACL reconstruction, leveraging Sr-containing biomaterials to substantially ameliorate ligament-bone healing. This advancement is poised to enhance clinical outcomes for patients undergoing ACL reconstructive procedures, offering a significant stride forward in the domain of orthopedic sports medicine (Stanton et al., 2022).

##### 5.1.3.3 Bone-ligament-bone integrated scaffold

The study presents the innovative design and development of a multiphasic bone-ligament-bone (BLB) integrated scaffold, specifically engineered for ACL reconstruction. This advanced scaffold is composed of polylactic acid (PLA) and deferoxamine (DFO) encapsulated within mesoporous hydroxyapatite (MHA) through a thermally induced phase separation (TIPS) process, complemented by silk fibroin (SF) and connective tissue growth factor (CTGF) embedded within Poly (L-lactide-co-ε-caprolactone) (PLCL) nanofiber yarns, crafted into a braided configuration. The mechanical properties of the BLB scaffold were meticulously assessed and confirmed to align with those of human ACLs, indicating its potential for effective clinical use. *In vitro* studies demonstrated that CTGF successfully stimulated the expression of genes vital for ligament formation, while the TIPS scaffolds, enriched with MHA and DFO, significantly enhanced osteogenic gene expression within bone marrow stem cells (BMSCs). These scaffolds also fostered the migration and tubular formation of human umbilical vein endothelial cells (HUVECs), indicating a promising capacity to support vascularization.

Within a rabbit model, the BLB scaffold proved instrumental in promoting ligamentization and graft-bone integration, effectively delivering bioactive substances to the repair site. The synergistic effect of dual delivery of DFO and calcium ions by the BLB scaffold was particularly noteworthy, as it significantly advanced bone regeneration. Concurrently, CTGF contributed to enhanced collagen formation and accelerated ligament healing, underscoring the scaffold’s multifaceted approach to ACL reconstruction and its potential to set a new benchmark in orthopedic biomaterials (Xie et al., 2024).

##### 5.1.3.4 Polycaprolactone/hydroxyapatite/collagen-based fiber scaffold

This study endeavored to ascertain the characteristics of an anterior cruciate ligament (ACL) scaffold composed of polycaprolactone (PCL), hydroxyapatite (HA), and collagen, across a spectrum of weight ratios: (50:45:5), (50:40:10), (50:35:15), (50:30:20), and (50:25:25). All specimens exhibited an average fiber diameter beneath the 1,000 nm threshold, signifying a nanoscale structure. Prominently, the scaffold with a 50:45:5 PCL:HA:collagen ratio demonstrated the most favorable attributes, including an average fiber diameter of 488 ± 271 nm. In terms of mechanical performance, the braided samples showcased an ultimate tensile strength (UTS) of 2.796 MPa and a modulus of elasticity of 3.224 MPa. In contrast, the non-braided counterparts possessed a UTS of 2.864 MPa and a notably higher modulus of elasticity at 12.942 MPa. The scaffolds were projected to degrade over an estimated period of 9.44 months. Furthermore, cytotoxicity evaluations confirmed the non-toxic nature of the samples, with a robust cell viability rate of 87.95%. These findings underscore the potential of the optimized 50:45:5 PCL:HA:collagen scaffold as a viable candidate for ACL regeneration applications (Aminatun et al., 2023).

#### 5.1.4 HA treatment limitations

While HA therapy presents a promising avenue for enhancing the healing of the ACL and restoring joint homeostasis, several limitations and challenges must be critically acknowledged and addressed to fully harness its therapeutic potential:

1) Limited Residence Time and Bioavailability: A primary challenge with HA treatment is its transient residence time *in vivo*, which may constrain its capacity to provide a prolonged therapeutic impact. To surmount this obstacle, researchers are investigating various strategies, including the administration of repeated injections, the development of slow-release formulations, and the use of HA-loaded stents, all aimed at prolonging HA’s duration of action and bolstering its regenerative capabilities (Ong et al., 2021).
2) Variable Molecular Weight and Composition: The efficacy of hyaluronic acid is contingent upon its molecular weight, the extent of its crosslinking, and the purity of the preparation. The standardization of HA products and the optimization of their physicochemical characteristics are paramount to ensuring reliable and uniform treatment outcomes (Mao et al., 2023).
3) Insufficient Clinical Efficacy Evidence: Despite the positive findings from preclinical studies on HA’s role in ACL healing, robust clinical evidence supporting its application in clinical practice is scarce. There is a need for well-designed, placebo-controlled, randomized clinical trials with substantial sample sizes and long-term follow-up periods to firmly establish the clinical efficacy of HA in the treatment of ACL injuries (Ong et al., 2021).
4) Potential Interactions with Other Therapies: In exploring HA as a potential therapy for ACL injuries, it is imperative to consider the local joint microenvironment, where HA can interact with other bioactive substances. These interactions may alter the therapeutic efficacy or safety of HA. Therefore, a comprehensive understanding of these potential interactions and the development of strategies to mitigate any adverse effects are crucial for safely and effectively integrating HA into a comprehensive, multimodal treatment plan. As previously mentioned, following ACL injury, the concentration of MMP-3 in the joint cavity fluid increases. MMPs are a group of enzymes that play a pivotal role in the degradation and remodeling of the ECM. The accumulation of intra-articular MMP-3 may hinder the healing process of the ACL, as they can degrade various ECM components, including HA (Wan et al., 2021). TNF-α is a significant pro-inflammatory cytokine that activates immune cells, such as macrophages and neutrophils. These cells release a variety of enzymes, including MMPs, during the inflammatory response, promoting the degradation of HA. Moreover, TNF-α may have synergistic effects with other inflammatory factors like IL-1β and IL-6, which could enhance the expression and activity of MMPs, further affecting the stability and functionality of HA (Xiao et al., 2021). To optimize the therapeutic potential of HA, the inflammatory joint microenvironment must be taken into account, and it may be necessary to modulate these inflammatory pathways through combination therapy or other interventions to protect HA from premature degradation, thereby supporting the recovery process of ACL injuries while minimizing potential adverse reactions.
5) Cost-Effectiveness and Accessibility: The financial implications of HA-based therapies, particularly when integrated with other advanced treatment modalities such as stents or serial injections, may present a barrier to their broad adoption. A thorough evaluation of the cost-effectiveness of hyaluronic acid therapy in comparison to traditional treatments, coupled with efforts to reduce expenses without sacrificing therapeutic efficacy, is vital for its consideration as a routine clinical intervention (Wan et al., 2021).

### 5.2 Stem cell therapy

#### 5.2.1 Mesenchymal stem cells (MSCs)

In the contemporary biomedical landscape, mesenchymal stem cells (MSCs), characterized by their pluripotency, self-renewal capacity, and ability to differentiate into a spectrum of cell types, have emerged as a focal point of regenerative medicine. Their therapeutic potential has been extensively substantiated through rigorous evaluation in both animal models and human clinical studies. MSCs have been shown to exert salutary effects on angiogenesis, cell proliferation, and the modulation of inflammatory responses, in addition to the secretion of a myriad of bioactive molecules that are integral to tissue repair mechanisms (Marolt Presen et al., 2019; Ding et al., 2023).

Recent scholarly work has suggested that the therapeutic efficacy of MSCs in the treatment of ACL injuries may be predominantly facilitated through the release of exosomes (Lui, 2021; Zou et al., 2023). Mesenchymal stem cell-derived exosomes (MSC-exos) are extracellular vesicles of nanometer dimensions that serve as conduits for intercellular communication. These vesicles are capable of transporting a constellation of biologically active molecules, including vascular endothelial growth factor (VEGF), basic fibroblast growth factor (bFGF), angiopoietin-1, monocyte chemotactic protein-1, interleukin-6 (IL-6), and placental growth factor, thereby exerting regulatory effects on the physiological and pathological processes within recipient cells (Chen et al., 2023; Liu et al., 2022; Matsuzaka and Yashiro, 2022; Yao et al., 2023).

In the context of ACL injury repair, the processes of angiogenesis and fibroblast proliferation are often impeded by a relative dearth of stem cells. The introduction of MSCs may serve to bolster these processes effectively and artificially, thereby augmenting the repair of damaged tissue. Importantly, MSCs contribute not only to the healing processes but also to the restoration of ligamentous integrity through their interactions with the osseous interface (Zou et al., 2023).

**FIGURE 3:**
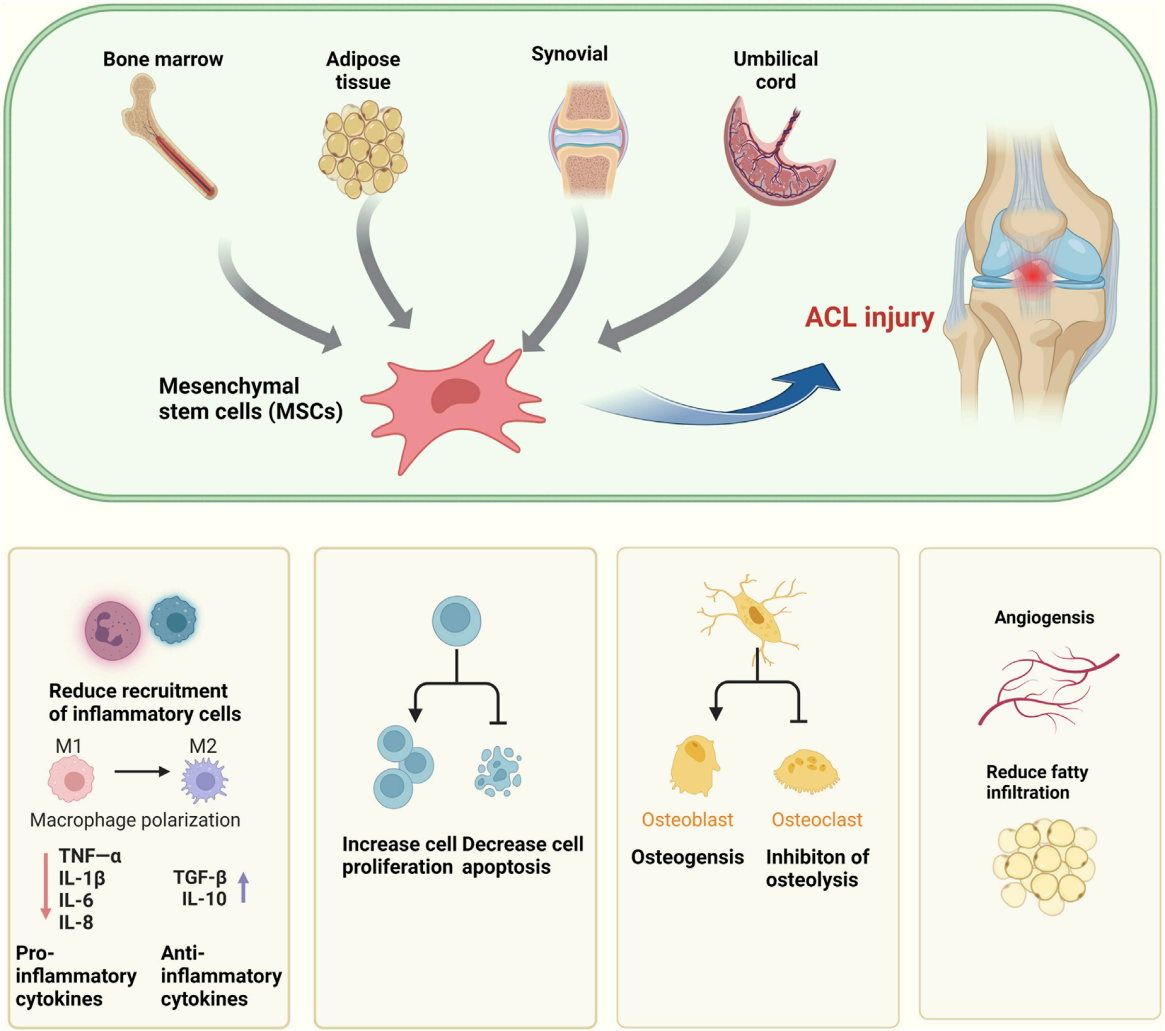
Application of MSCs in the treatment of ACL.

This line of inquiry presents a compelling array of novel therapeutic strategies for the management of ACL injuries, offering a promising avenue for the advancement of clinical treatment protocols. The harnessing of MSCs and their exosomes represents a paradigm shift in regenerative medicine, with the potential to markedly enhance the clinical outcomes for patients afflicted with ACL injuries (Figure 3).

##### 5.2.1.1 MSCs animal models

In the domain of animal studies focused on ACL injury, both MSCs and hematopoietic stem cells have demonstrated notable therapeutic potential. These cellular agents secrete a cadre of pivotal growth factors that exert positive influences on the reparative processes associated with ACL injury (Zhu et al., 2023a; Rhatomy et al., 2023). Illustratively, in a series of rat studies, whole bone marrow cells (BMCs), which were enriched with MSCs and hematopoietic stem cells, were intra-articularly injected into the joint cavity. This intervention led to a significant elevation in the levels of TGF-β1 within the knee joint fluids, thereby fostering an environment conducive to ACL healing (Park et al., 2021).

Building upon these findings, Sun et al. (2016) conducted research wherein rabbit MSCs were subjected to mechanical stimulation via radial strains at a frequency of 0.1 Hz. This mechanical stimulation was found to enhance both the proliferation and collagen production of the MSCs. In a complementary study, porcine MSCs were isolated from retro-patellar fat pads, denoted as adipose-derived stem cells (ADSCs), and from peripheral blood mononuclear cells (PBMCs). These cells were subsequently co-cultured with porcine ACL fibroblasts. The ADSCs were observed to stimulate both the proliferation and collagen production in ACL fibroblasts, while the PBMCs facilitated the migration of ligament fibroblasts. Under conditions of co-culture with ADSCs and ACL fibroblasts, there was a marked increase in collagen gene expression (Proffen et al., 2013) (Table 1).

**TABLE 1:** Different stem cell therapies used to biologically treat anterior cruciate ligament (ACL) injuries.

Animal models	Cell type	Effects	References
Rat	MSCs and BMCs	Joint cavity MSC injections with elevated local TGF-β1 levels to promote ACL healing	Park et al. (2021), Fan et al. (2022)
	TDSCs	TDSCs promote cell proliferation and ACL healing	Salingcarnboriboon et al. (2003)
Rabbit	MSCs	MSCs promote cell proliferation and collagen production	Sun et al. (2016)
Pig	MSCs	MSCs and ACL fibroblast co-culture promote cell proliferation, migration, and collagen production	Proffen et al. (2013)
Human	MSCs	Significant improvements in damaged ACL integrity	Centeno et al. (2018)
	BMCs	Significant improvements in injured ACL integrity, decreased pain visual analog scale scores, and increased lower extremity functional scale scores	Moraes et al. (2013)
	CD34+SCs	CD34+SCs express VEGF, promote ligament healing and regeneration, angiogenesis, and improves biomechanical strength in injured ACLs	Matsumoto et al. (2012), Mifune et al. (2013), Dun et al. (2023)
	ADSCs	ADSCs differentiate into ligament cells with low HLA effects	Qin et al. (2023)
	TDSCs	Higher proliferation rates and *in vitro* osteogenic differentiation capacity than MSCs	Chen et al. (2023), Kim et al. (2022)

Abbreviations: MSCs, mesenchymal stem cells; TGFβ1, transforming growth factor β1; ACL, anterior cruciate ligament; BMCs, bone marrow cells; TDSCs, tendon and ligament-derived stem and progenitor cells; CD34+SCs, CD34+expressing stem cells; ADSCs, adipose-derived stem cells; HLA, human leukocyte antigen.

Collectively, these studies underscore the significant biological roles that MSCs and their associated cell types play in the repair of ACL injuries. Furthermore, they offer a burgeoning foundation for the development of innovative therapeutic strategies aimed at enhancing the clinical outcomes for patients suffering from ACL injuries. The insights gleaned from these preclinical investigations pave the way for future research endeavors and potential clinical applications, marking an exciting frontier in the field of orthopedic regenerative medicine.

##### 5.2.1.2 MSCs in human ACL repair

In the quest to delineate the clinical efficacy of MSCs in the treatment of human ACL injuries, researchers have employed *in situ* methodologies to harvest stem cells from the damaged ACL stumps of affected patients. Post isolation, these cells undergo a meticulous characterization process, during which they are identified as MSCs based on a constellation of criteria including their adherence properties, distinctive morphological characteristics, specific surface marker expression, and their capacity for multilineage differentiation (Yin et al., 2023; Knapik et al., 2021). These observations collectively imply that the isolated stem cells may serve as viable candidates for autologous cell sources in the regeneration of the ACL.

In a pivotal study conducted by Centeno et al. (2018), patients presenting with ACL tear injuries were administered percutaneous injections of ACL-derived MSCs. The therapeutic outcomes were subsequently assessed using magnetic resonance imaging (MRI), a non-invasive diagnostic tool employed to quantify pixel density as an objective metric of ACL integrity. The study demonstrated significant enhancements in ACL integrity following MSC treatment, thereby positing this treatment modality as a promising non-surgical alternative for ACL injury management. These findings contribute to the burgeoning body of evidence suggesting that MSC-based therapies may offer a less invasive and equally effective approach to ACL repair, potentially transforming the standard of care for patients with ACL injuries (Table 1).

#### 5.2.2 BMCs

BMC injections represent an effective biotherapeutic strategy for the treatment of partial ACL injuries. This approach circumvents the laborious process of systematically collecting and culturing MSCs, thereby enhancing its clinical feasibility. Furthermore, cells differentiated from hematopoietic stem cells secrete a spectrum of key growth factors and cytokines that actively contribute to the ACL healing process. In rat models, BMC injections have been associated with elevated levels of TGF-β1, suggesting a mechanism by which BMCs may promote ACL healing through the secretion of growth factors (Fan et al., 2022). Consequently, BMC injections are posited as a straightforward and efficient therapeutic modality, offering novel possibilities for the management of partial ACL injuries.

Despite their therapeutic potential, BMCs necessitate further investigation to address several challenges before achieving widespread clinical application. Firstly, ACL injuries encountered in clinical practice may differ significantly from those in controlled laboratory rat models. The BMC injection method appears more applicable to partial ACL injuries, with complete ACL tears potentially requiring surgical reconstruction, albeit potentially augmented by BMC therapy. Secondly, the optimal concentrations and dosages of BMCs for human administration remain undefined. In a study by Moraes et al. (2013), precise BMC injections into injured patient ACLs, guided by fluoroscopy, demonstrated promise for treating Grade 1, 2, and possibly Grade 3 ACL lacerations (in the absence of traction). Magnetic resonance imaging (MRI) studies corroborated these findings, with improvements in ACL integrity and a reduction in patient pain visual analog scale scores, alongside an increase in lower extremity functional scale scores (Table 1). These results provide significant clinical evidence supporting the use of BMCs in the management of ACL injuries.

#### 5.2.3 ADSCs

Adipose-derived stem cells (ADSCs) have garnered significant attention in regenerative medicine due to their unique biological properties and numerous practical benefits. One of the most notable advantages of ADSCs is their ability to be easily and safely isolated from a patient’s adipose tissue through minimally invasive procedures, such as liposuction. This accessibility makes ADSCs a viable and appealing cell source for a diverse patient population, regardless of age or health condition (Song et al., 2023; Dong et al., 2023).

ADSCs are highly versatile, with the ability to differentiate into various tissue types, including adipose, muscle, cartilage, and fibroblasts, making them ideal for treating a wide range of conditions, from degenerative diseases to traumatic injuries. Additionally, compared to BMCs, ADSCs express lower levels of human leukocyte antigen I (HLA-I), which significantly reduces the risk of immune rejection in allogeneic therapies (Qin et al., 2023).

Their enhanced immunomodulatory capacity and low immunogenicity further position ADSCs as an ideal choice for clinical applications that involve immune-related treatments, such as managing autoimmune disorders, graft-versus-host disease (GVHD), and improving organ transplantation outcomes.

In conclusion, the ease of isolation, multipotency, low immunogenicity, and robust immunosuppressive properties of ADSCs make them an especially attractive option for advancing regenerative medicine, particularly in therapies that require careful immune system management.

#### 5.2.4 CD34+ stem cells (CD34+ SCs)

Matsumoto et al. made groundbreaking advancements in treating patients undergoing arthroscopic primary ACL reconstruction by identifying CD34+ stem cells (SCs) within damaged ACL tissues. These cells demonstrated the ability to differentiate into various cell types and migrate to ACL fracture sites, significantly contributing to the healing process. Building on this discovery, the team developed an innovative tissue-engineered contractile cell sheet technique, wrapping grafts with CD34+ SCs during ACL reconstructive surgery. This approach restored proprioception, accelerated graft maturation, and enhanced biomechanical strength while promoting the healing of the osteotendinous junction and graft ligament (Matsumoto et al., 2012; Mifune et al., 2013).

A key finding was that CD34+ SCs expressed vascular endothelial growth factor (VEGF), which stimulated angiogenesis, crucial for improving the biomechanical integrity of the repaired ACL. This pioneering method not only advanced surgical practice but also offered a new strategy in ACL injury treatment by integrating regenerative medicine and stem cell technology (Dun et al., 2023). The implications of these findings are profound, paving the way for improved outcomes in ACL reconstruction. The use of CD34^+^ SCs in grafts enhances structural repair and supports a more comprehensive healing process, potentially reducing recovery time and improving long-term knee function. Matsumoto et al.’s research represents a significant leap in ACL treatment, laying the groundwork for future applications of regenerative approaches in orthopedic surgery.

#### 5.2.5 Tendon and ligament-derived stem and progenitor cells (TDSCs)

In 2003, researchers made a major breakthrough by isolating pluripotent stem cells from mouse tendons, which were similar to mesenchymal stem cells (MSCs) and capable of differentiating into adipocytes, osteoblasts, and chondrocytes (Salingcarnboriboon et al., 2003). This discovery paved the way for further research that identified similar stem cells within the human anterior cruciate ligament (ACL). These ACL-derived stem cells demonstrated superior proliferation rates and osteogenic differentiation capacities compared to traditional MSCs (Chen et al., 2023; Kim et al., 2022) (Table 1).

The identification of these ACL-specific stem cells has significant implications for regenerative medicine. Their ability to rapidly proliferate and differentiate offers new possibilities for developing advanced therapies for ACL injuries, potentially improving healing and outcomes for patients. This research has also expanded our understanding of the regenerative potential within tendons and ligaments, opening new avenues for tissue engineering and the development of innovative treatments for ligament repair.

In summary, the discovery of stem cells within tendons and the human ACL marks a pivotal advancement in the field, offering promising new strategies for treating ACL injuries and enhancing the prospects of regenerative medicine and tissue engineering.

#### 5.2.6 Stem cell therapy limitations

While stem cell therapy has shown remarkable biological safety profiles, potential carcinogenicity and cancer resistance risks persist (Lu et al., 2021). Autologous isolated stem cells have little risk of immune rejection or inflammatory reactions, which are significant advantages. However, stem cells from other donors carry higher risks of immune rejection. Additionally, long-term stem cell therapy side effects remain unclear and include the possibility of attacking normal cells in recipients, thus causing bleeding and infection issues. Another critical area is how to ensure that stem cells are delivered to the right location at the right concentration to achieve effective cell proliferation and tissue regeneration while avoiding unwanted scar tissue formation (Sinkler et al., 2023; Krawetz et al., 2023). Stem cell therapy costs are also very high, which limits its popularity, especially in resource-limited settings. Therefore, while stem cell therapy shows great therapeutic potential in many aspects, it faces considerable challenges and limitations in terms of clinical applications, and more research and clinical trials are required to overcome these problems.

### 5.3 Growth factor therapy

In the early stages of ACL injury healing, multiple growth factors play crucial roles in the ligament repair process. These factors are vital signaling molecules that regulate tissue repair and regeneration, making them key players in initiating and guiding the healing response.

Growth factor therapy has become a popular approach for treating ACL injuries, leveraging the natural reparative properties of these molecules to enhance the body’s healing mechanisms. Research shows that growth factors promote cell proliferation, which is essential for replenishing damaged cells and supporting tissue repair. They also facilitate angiogenesis, ensuring an adequate blood supply to the injured ligament, which is crucial for the survival and function of regenerating tissue (Sha et al., 2022).

Moreover, growth factors play a significant role in remodeling the extracellular matrix (ECM), which provides structural support to the ligament. By promoting collagen synthesis and proper ECM organization, growth factors help restore the ligament’s strength and flexibility, making it more resilient to future injuries. Additionally, they enhance cell adhesion, ensuring the structural cohesion of the healing ligament.

**FIGURE 4:**
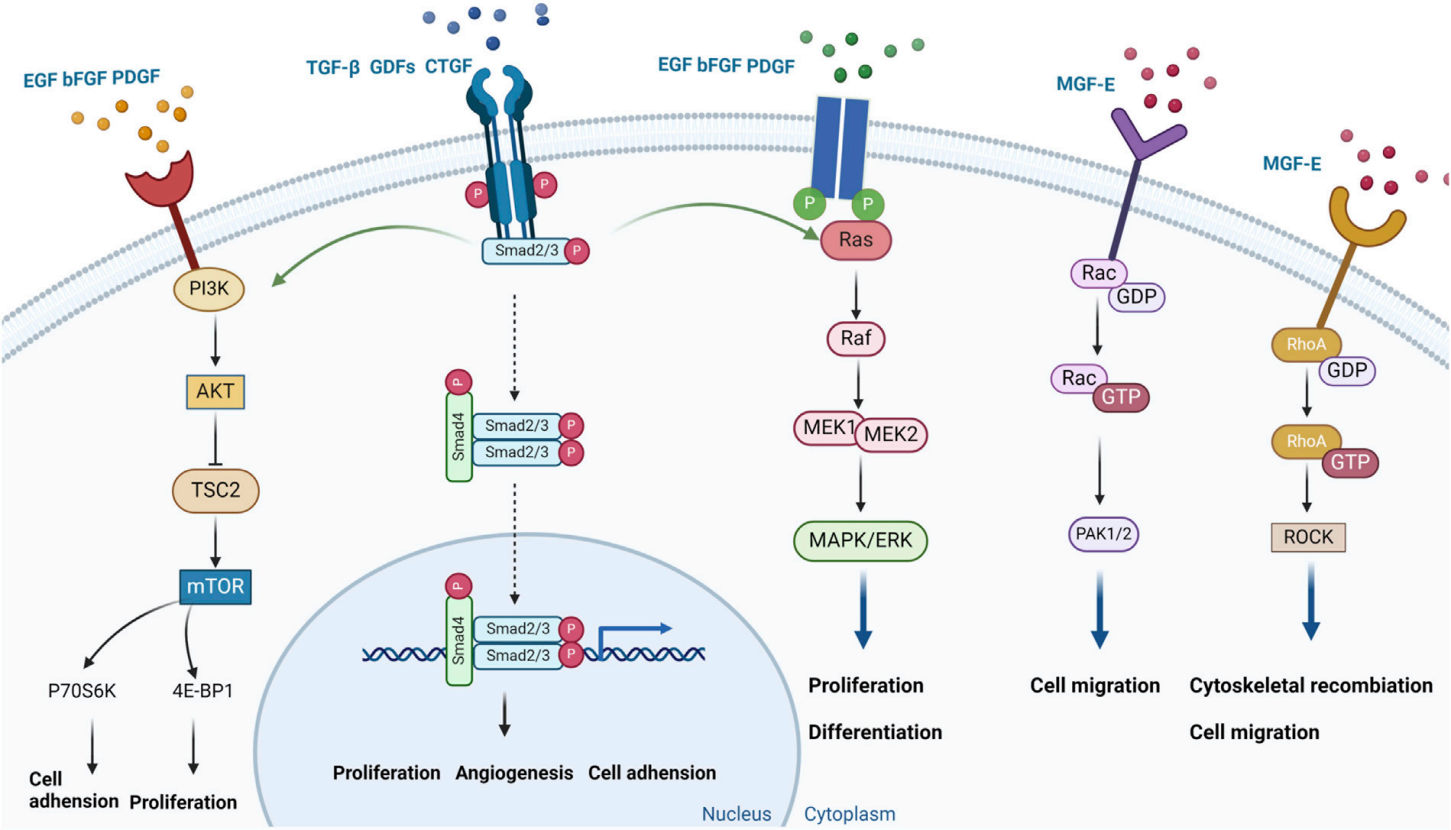
Growth factors signaling pathway in the treatment of ACL injury.

These findings underscore the potential of growth factor therapy as a powerful tool in ACL injury treatment. As research advances, more effective therapeutic strategies are likely to emerge, improving recovery outcomes for patients and advancing regenerative medicine in orthopedics (Figure 4).

#### 5.3.1 Epidermal growth factor (EGF)

Upon binding to the epidermal growth factor receptor (EGFR), epidermal growth factor (EGF) triggers the receptor’s tyrosine kinase activity, thereby initiating a cascade of kinases within the mitogen-activated protein kinase (MAPK) pathway. This includes the sequential activation of RAF, MEK, and ERK. The phosphorylated ERK translocates to the nucleus, where it phosphorylates a variety of transcription factors, thereby promoting the expression of genes associated with cell proliferation. The activation of EGFR also stimulates the phosphatidylinositol-3 kinase (PI3K) pathway, leading to the activation of AKT. The signaling pathway plays a pivotal role in cell proliferation and the progression of the cell cycle (Yuan et al., 2022). In rabbit ACL studies, the EGF not only significantly increased ACL fibroblast proliferation, but also enhanced protein synthesis which helped prevent mechanical property decline in damaged ACLs. This was possibly because EGF applications may have affected collagen cross-linking properties; however, further studies are required to gain a deeper understanding of collagenase activity and collagen network cross-linking characteristics in ACLs (Gui et al., 2022; Zhu et al., 2023b). In McKean et al., human ACL fibroblasts were cultured by dissecting ACLs from adult male cadavers (6–24 h after death). It was observed that EGF had significant effects on human ACL-derived fibroblast adhesion and proliferation (McKean et al., 2004). However, direct injections of natural growth factors into damaged sites in patients may require continuous injections due to their rapid actions and short duration times. Other studies have reported that immobilized cytokines can act on high molecular weight substrate surfaces without depleting cytokines. For example, photo-immobilized EGF was relatively and rapidly induced and sustained human ACL fibroblast proliferation via an artificial stimulation system (Woo et al., 2007) (Table 2). These findings provide new insights and approaches for EGF use in ACL injury therapy, especially when maintaining long-lasting EGF effects. Such approaches may lead to more effective therapeutic strategies when biologically treating ACL injuries.

**TABLE 2:** Growth factor types and their effects.

Signaling molecules	Animal models	Effects	References
EGF	Rabbit	Stimulates fibroblast proliferation, collagen and non-collagen synthesis and improves mechanical properties	Gui et al. (2022), Zhu et al. (2023a)
	Human	Stimulates fibroblast adhesion in ACL	McKean et al. (2004)
EGF treated by photoimmobilization	Human	Rapid and sustained proliferation of human ACL fibroblasts induced by an artificial near-side stimulation system	Woo et al. (2007)
bFGF	Rat	Enhances cell proliferation, wound re-epithelialization, collagen deposition, and contraction, without any noticeable toxicity and inflammation	Zhang et al. (2018), Savitri et al. (2024)
bFGF	Rabbit	Stimulates fibroblast proliferation in ACL	Scherping et al. (1997), Date et al. (2010)
bFGF	Human	Stimulates DNA synthesis	Yoshida and Fujii, (1999)
PDGF	Rat	Increases the secretion of VEGF to promote angiogenesis	Petersen et al. (2003)
PDGF-BB	Rabbit	Enhances the migratory ability of ACL cells	Petersen et al. (2003)
PDGF-BB	Rabbit	Stimulates fibroblast proliferation	Scherping et al. (1997)
MGF-E peptide	Human	Stimulates fibroblast proliferation	Sha et al. (2022)
MGF-E peptide	Human	Protects fibroblasts from hypoxia-induced apoptosis; promotes fibroblast proliferation; promotes angiogenesis	Sha et al. (2019)
GDF-5	Rabbit	Stimulates fibroblast proliferation	Date et al. (2010)
GDF-7	Rabbit	Promotes fibroblast proliferation and increases collagen 1 expression	Date et al. (2010)
GDF-8	Rat	Inhibits osteogenic differentiation and the proliferation of chondrocytes	Brashear et al. (2021)
Anti-GDF8 antibodies	Rat	Prevents musculoskeletal degeneration following joint injury	Brightwell et al. (2023)
CTGF	Rabbit	Promotes healing in injured ligamentous tissue	Zhang et al. (2017), Sun et al. (2017)
TGF-β	Human	Stimulates DNA synthesis but does not increase collagen synthesis	Yoshida and Fujii, (1999)
TGF-β	Human	Promotes extracellular matrix synthesis, affect cell differentiation, and activates Smad cell signaling pathway	Morita et al. (2021)
TGF-β	Sheep	Promotes early ACL injury healing in sheep	Spindler et al. (1996)
TGF-β	Rabbit	Stimulates collagen and non-collagen synthesis and improves mechanical properties	Sakai et al. (2002)

Abbreviations: EGF, epidermal growth factor; ACL, anterior cruciate ligament; bFGF, basic fibroblast growth factor; PDGFBB, platelet-derived growth factor-BB; MGF-E, mechano growth factor E peptide; GDF, growth differentiation factor; CTGF, connective tissue growth factor; TGF-β, Transforming growth factor-β.

#### 5.3.2 Basic fibroblast growth factor (bFGF)

Basic fibroblast growth factor (bFGF) initiates a signaling cascade by binding to fibroblast growth factor receptors (FGFR), thereby activating the receptor’s tyrosine kinase activity. This activation leads to the sequential engagement of kinases within MAPK pathway, notably MEK and ERK. Once activated, ERK enters the cell nucleus, phosphorylating a plethora of transcription factors, which in turn, augments the expression of genes vital for cell proliferation. Furthermore, bFGF, through its interaction with FGFR, can initiate the PI3K pathway, culminating in the activation of AKT. This signaling network is integral in modulating cellular processes such as survival and proliferation (McKean et al., 2004). bFGF significantly promoted rabbit ACL fibroblast proliferation (Scherping et al., 1997; Date et al., 2010). Also, Yoshida and Fujii (1999) observed that bFGF stimulated DNA synthesis in human ligament cells, but no significant collagen expression changes were reported (Table 2). Further studies also showed that the 17 kDa bFGF peptide form was a major promoter of cell proliferation and was expressed in wound healing and fracture repair applications. However, under normal physiological conditions, bFGF may rapidly lose its biological activity if not stabilized. To improve its efficiency, some bFGF studies have integrated the molecule into hydrogels that release drugs in a sustained manner. This approach not only improved bFGF stability, but also enhanced its therapeutic effectiveness. Research has found that integrating bFGF into hydrogels, the bioinspired hydrogels containing bFGF can significantly enhance cell proliferation, wound re-epithelialization, collagen deposition, and contraction, without any noticeable toxicity and inflammation. However, the specific methods and effects of using them to treat ACL injuries require further exploration (Zhang et al., 2018; Savitri et al., 2024) (Table 2). Nevertheless, these findings emphasize the importance of bFGF in the treatment of ACL injuries and highlight the need for further research to improve its stability and effectiveness. Therefore, bFGF has the potential to become a key factor improving outcomes in treating ACL injuries.

#### 5.3.3 Platelet-derived growth factor (PDGF)

Platelet-derived growth factor (PDGF) is a key mitogen in serum, exerting a significant activating effect on cells of mesenchymal origin. PDGF plays an important role in the treatment of ACL injuries, with its main therapeutic pathways including the promotion of cell proliferation and migration, and the activation of various signaling pathways involved in the remodeling of the extracellular matrix and tissue repair processes. PDGF exists in three different heterodimer forms, including AA, BB, and AB heterodimers, which exert biological effects by binding to specific receptors (Meyer-Ingold and Eichner, 1995; Zhang et al., 2023). Upon binding to its receptor PDGFR, the receptor dimer undergoes autophosphorylation, activating its tyrosine kinase activity and signaling pathways including PI3K/AKT and MAPK/ERK. These pathways promote the expression and activity of cell cycle-related proteins, such as cyclin-dependent kinases (CDKs) and cyclins, driving cells from the G1 phase into the S phase for DNA replication. PDGF is crucial for cell survival, proliferation, differentiation, and migration (Zou et al., 2022; Contreras et al., 2021). Current research primarily focuses on PDGF-BB for promoting healing of ACL injuries, as the ACL does not show a significant proliferative response to PDGF-AA or PDGF-AB (Spindler et al., 1996; Scherping et al., 1997). Research by Scherping et al. indicates that PDGF-BB significantly affects the proliferation of rabbit ACL fibroblasts, suggesting an important application of PDGF in facilitating ligament healing. PDGF not only directly promotes cell proliferation but may also indirectly promote tissue repair and regeneration by affecting the synthesis and degradation of the extracellular matrix, angiogenesis, and cell migration (Scherping et al., 1997). Studies have found that after a 12-h incubation in a 1% FBS medium, 1 ng/mL of PDGF-BB significantly enhances the migratory ability of ACL cells, which may aid in the healing process of ACL injuries (Kobayashi et al., 2000). Research using rat achilles tendon fibroblasts has shown that PDGF significantly increases the secretion of VEGF by tendon cells under hypoxic conditions, demonstrating a synergistic effect that is crucial for angiogenesis and early tissue repair during ACL remodeling (Petersen et al., 2003). Given PDGF’s multiple roles in promoting cell proliferation, migration, and angiogenesis, it holds broad application prospects as a potential biotherapeutic target in the treatment of ACL injuries (Chen et al., 2022). In summary, PDGF demonstrates potential in ACL injury treatment through its promotion of proliferation, migration, and angiogenesis in ACL fibroblasts, as well as its activation of signaling pathways. Future research will continue to explore the application of PDGF in tissue engineering and bio-scaffold design to achieve more effective ACL injury treatment and recovery (Table 2).

#### 5.3.4 Force growth factor E peptide

Research by Sha et al. (2019) demonstrated that myogenic growth factor (MGF) E peptide could be used for human ACL repair and regeneration. ACL fibroblasts undergo apoptosis in severely hypoxic microenvironments. The MGF E peptide effectively protects ACL fibroblasts from hypoxia-induced apoptosis by regulating caspase 3/7/9 mRNA and related apoptotic protein expression levels while promoting cell proliferation. MGF also recruits vasculopoietic cells via the stromal cell-derived factor 1 alpha/CXCR4 axis to promote angiogenesis-generating cells and stimulate VEGFα expression, thereby promoting angiogenesis after ACL damage. It was reported that MGF promoted damaged ACL fibroblast proliferation by targeting Rac1-PAK1/2 and RhoA-ROCK1 signaling pathways (Sha et al., 2022) (Table 2). These findings highlight multiple MGF roles in treating ACL injury, including fibroblast protection, promoting cell proliferation and angiogenesis, and activating repair-related signaling pathways. Thus, MGF E peptides may provide new and promising biotherapeutic possibilities for treating ACL injuries.

#### 5.3.5 Growth differentiation factors (GDFs)

Growth and differentiation factors (GDFs) have been identified as key signaling molecules in the embryonic development of tendons. In conjunction with TGF-β, BMPs, and FGFs, GDFs are instrumental in the embryonic genesis of tendon cells. The majority of joint tissues derive from GDF5-positive mesenchymal cells, potentially contributing to the formation of the ACL. GDFs are conjectured to possess a proteogenic role in tendon development, possibly triggering cytoskeletal reorganization or the Smad signaling cascade (Wang et al., 2021). GDF-5 and GDF-7 have demonstrated the ability to stimulate the proliferation of rabbit ACL fibroblasts. Significantly, GDF-5 has been shown to markedly enhance the expression of COL1 in ACL fibroblasts (Date et al., 2010) (Table 2). This discovery implies that GDF-5 and GDF-7 are of considerable importance in the facilitation of ACL healing and regeneration. Specifically, GDF-5, by boosting COL1 expression, can aid in the restoration of structure and function in the damaged ACL. GDF8, also recognized as myostatin, is implicated in the etiology of musculoskeletal degeneration subsequent to ACL injury. GDF8 functions as an inhibitor of muscle mass and initiates Smad2/3 signaling, influencing transcriptional activity. Beyond promoting muscle atrophy, it also curbs osteogenic differentiation and the proliferation of chondrocytes (Brashear et al., 2021). Research has illustrated that the inhibition of GDF8 in murine models can lead to the recovery of muscle mass and functionality, as well as a decrease in the intra-articular expression of MMPs. GDF8 emerges as an early modifiable target post-ACL injury, with the administration of anti-GDF8 antibodies presenting a promising therapeutic approach against post-joint injury musculoskeletal degeneration (Brightwell et al., 2023). Consequently, these GDFs may act as prospective biotherapeutic agents, potentially enhancing treatment strategies for ACL injuries.

#### 5.3.6 Connective tissue growth factor (CTGF)

Connective tissue growth factor (CTGF), a member of the TGF-β superfamily, acts in concert with TGF-β to activate both Smad-dependent and Smad-independent signaling pathways. CTGF has key roles in damaged rabbit ACL healing processes and exerts its effects by upregulating COL1 and COL2 expression. This finding has provided more CTGF possibilities in treating injured tissues and promoting wound healing (Zhang et al., 2017; Sun et al., 2017) (Table 2). CTGF promotes collagen expression, which is a major fibrous component of the extracellular matrix in connective tissues, and is essential for maintaining ACL structural integrity and function. Therefore, CTGF, as a promising biotherapeutic, may be used for ACL injury treatment and recovery. These findings highlight new research directions for the biotherapeutic treatment of ACL injury and regenerative medicine in general.

#### 5.3.7 Transforming growth factor-beta (TGF-β)

Transforming growth factor-beta (TGF-β) is a pleiotropic cytokine that plays a crucial role in cell growth, differentiation, apoptosis, tissue repair, and immune modulation. TGF-β promotes the synthesis of extracellular matrix proteins, such as collagen and fibronectin, which are major components of the cellular microenvironment. During wound healing and tissue repair, TGF-β is instrumental in facilitating cell proliferation, migration, and differentiation, as well as the synthesis of the extracellular matrix. By binding to its receptors, TGF-β activates a cascade of signaling pathways, including both Smad-dependent and Smad-independent routes (Wang et al., 2021).

In sheep ACLs, TGF-β was reported to potentially promote initial healing processes in damaged ligaments (Spindler et al., 1996). In rabbit ACL studies, TGF-β stimulated fibroblast proliferation and increased collagen and non-collagen synthesis in cells. Additionally, TGF-β prevented decreases in the mechanical properties of autologous tendons by significantly inhibiting increased water content in tendons, inhibiting cross distribution and elevated cross-sectional areas, and preventing decreases in tensile strength (Sakai et al., 2002). Yoshida and Fujii (1999) reported that TGF-β and bFGF stimulated DNA synthesis but did not collagen synthesis in human ligament cells (Table 2). These findings suggested that TGF-β may have important roles in both initial healing and long-term repair processes in ACL injury. In particular, its promotion of fibroblast functions is essential for maintaining and restoring ACL structural integrity and function. In the study by Morita et al. (2021), TGF-β was found to induce the expression of fibrosis-associated genes in tendon cells, such as Aggrecan (ACAN). TGF-β1 plays a pivotal role in tendon healing by promoting extracellular matrix synthesis, influencing cell differentiation, activating the Smad cell signaling pathway, and inducing a fibrotic phenotype. Therefore, TGF-β is a promising biotherapeutic in treating ACL injuries.

#### 5.3.8 Combinations of different types and doses of growth factors

Growth factors, as a class of polypeptides capable of regulating cell proliferation, differentiation, and matrix synthesis, play a crucial role in ligament tissue repair. Although direct injection of growth factors can provide immediate biological activity, their application is limited by several constraints. Firstly, the half-life of growth factors is typically short, which means frequent administration is required to achieve therapeutic effects. Secondly, direct injection may lead to non-specific distribution of growth factors in the body, increasing the risk of side effects. Additionally, the direct use of growth factors may elicit an immune response, affecting their efficacy and safety. To address these issues, gene therapy offers a promising alternative strategy. By utilizing gene therapy vectors, such as recombinant adeno-associated virus (rAAV) vectors, the genes encoding specific growth factors can be directly delivered into target cells, achieving localized and sustained overexpression of multiple growth factors. This approach not only extends the active time of growth factors in the body but also reduces systemic side effects and enhances therapeutic outcomes.

Different growth factors affect various cellular processes, such as cell proliferation, differentiation, migration, and matrix synthesis. Their combined use can simultaneously activate multiple biological pathways. Some growth factors may exhibit synergistic effects when used in combination, resulting in an overall effect greater than the sum of their individual effects. In a study by Chen et al., researchers infected BMSCs with adenoviral vectors carrying BMP2 and bFGF (AdBMP2 and AdbFGF), and then implanted these cells at the tendon-bone interface. They found that the combined therapy, through the activation of the Smad pathway, promoted fibrotic-related biological processes more effectively than monotherapy in facilitating tendon-bone healing and osteogenic differentiation after ACL injury (Chen et al., 2016). The overexpression of FGF-2 and TGF-β in hMSCs using rAAV vectors to promote ACL repair showed that the overexpression of these factors could enhance cell proliferation, increase DNA content, and promote the deposition of proteoglycans and collagen, contributing to improved quality and function of the repaired tissue (Stehle et al., 2024; Amini et al., 2023). The use of gene therapy vectors allows for precise control of the expression of combined growth factors, including their expression levels and duration, providing more flexibility for clinical treatment. Through gene-editing techniques, researchers can further optimize the gene sequences of growth factors to enhance their biological activity or improve their pharmacokinetic properties. This emerging technology of gene therapy combined with multiple growth factors requires further exploration of the optimal vector systems, gene-editing strategies, and administration routes in future research to achieve the best clinical outcomes of growth factor therapy.

#### 5.3.9 Growth factor therapy limitations

Although growth factors have demonstrated multiple potential effects in treating ACL injuries in terms of promoting cell proliferation, angiogenesis, and reducing apoptosis, other considerations must be accounted for when using these therapeutic approaches. As biologically active molecules, growth factors typically have short half-lives, which lead to critical questions regarding the duration of validity, optimal time points for treatment, and frequency of use as a therapy. Answering these vital questions will ensure that their therapeutic effects are maximized. For example, growth factor application time points may affect action efficiency in healing processes, while frequency of use must ensure therapeutic efficacy while avoiding possible side effects. While growth factor therapy provides promising treatment strategies for ACL injury, their clinical application requires more research and optimization.

### 5.4 Gene therapy

#### 5.4.1 Gene transfer vectors

Gene therapy, as a cutting-edge treatment, can sustainably and effectively promote ACL repair by transfecting therapeutic gene sequences which target ACL. Treatments may be conducted in two ways: by directly injecting vectors expressing therapeutic genes *in vivo*, or by genetically modifying cells or grafts *in vitro* and then reimplanting them into recipients (Amini et al., 2022). Gene therapy vectors are divided into two categories: non-viral and viral vectors. The latter category includes adenovirus, herpes simplex virus (HSV), retrovirus, lentivirus, and recombinant adeno-associated virus (rAAV) vectors. With their own unique advantages and limitations, choosing appropriate vectors is critical to ensure efficacious and safe gene therapy. Gene therapy for treating ACL injuries provides an innovative approach that promotes repair and regeneration by directly modifying genes in damaged tissues or cells. With continuous developments and improvements in this field, gene therapy has great potential for treating ACL injuries.

Non-viral vectors are relatively safe choices due to their non-replication and non-immunogenic characteristics. However, as they express genes during cell division (Zu and Gao, 2021; Gantenbein et al., 2020), gene transfer efficiencies are usually low, which is a major application limitation. In contrast, adenovirus and HSV vectors have higher transfer efficiency rates; they activate host immune responses and directly modify cells in division or quiescent phases (Goins et al., 2016; Greber and Gomez-Gonzalez, 2021; Watanabe et al., 2021). However, immune responses may pose safety concerns. Retroviral vectors integrate into host cell genomes, thus enabling long-term transgene expression. However, they only modify dividing cells, thereby limiting their application (Elsner and Bohne, 2017). RAAV vectors are considered safer alternatives, and efficiently modify dividing and quiescent cells over longer periods (Shirley et al., 2020; Verdera et al., 2020). When selecting gene therapy vectors, safety, efficiency, and applicability factors must be comprehensively considered to ensure treatment effectiveness and patient safety. With further research and technological advances, more effective and safer gene therapy vectors will be developed.

#### 5.4.2 Therapeutic factors

##### 5.4.2.1 Growth factors

Steinert et al. (2008) reported that after collagen hydrogel insertion into severed ACL ends, cells migrated from damaged ligaments into the hydrogel and began to repair tissue. This process was enhanced by transferring insulin-like growth factor-1 (IGF-1) cDNA into fibroblasts using adenoviral vectors to repair ACLs. In their *in vitro* studies, Haddad-Weber et al. (2010) demonstrated that by encoding cDNAs for BMPs 12 and 13 and after transduction through adenoviral vectors, they promoted collagen proliferation and secretion by fibroblasts. Thus, ACL fibroblast activity was enhanced and contributed to damaged ACL repair. Researchers (Wei et al., 2008; Goomer et al., 2000; Groth et al., 2017) constructed adenoviral vectors expressing TGF-β1 and VEGF165 genes. Co-expressed genes induced relatively rapid and sustained ACL fibroblast proliferation, increased COL1 and COL3 gene expression, and also fibronectin mRNA expression in rabbits, which exerted stronger and better effects on the migration and matrix synthesis of ACL fibroblasts. Madry et al. (2013) overexpressed FGF-2 using direct gene transfer mediated by rAAV vectors, which promoted human ACL injury healing. FGF-2 is a potent stimulator of fibroblast proliferation and type I/III collagen production. These studies showed that ACL repair and regeneration were effectively promoted by combining biomaterials and gene therapy techniques to treat ACL injuries (Table 3).

**TABLE 3:** Applications of gene therapy for ACL repair.

Vectors	Cell targets	Genes	References
Adenovirus	Fibroblast	IGF-1	Steinert et al. (2008)
Adenovirus	Fibroblast	BMPs 12 and 13	Haddad-Weber et al. (2010)
Adenovirus	Fibroblast	TGF-β1 and VEGF165	Wei et al. (2008)
Adenovirus	MSCs	SCX	Gulotta et al. (2011)
Adenovirus	MSCs	SCX, MKX	Otabe et al. (2015)
rAAV	Fibroblast	FGF-2	Madry et al. (2013)
Lentiviral vectors	MSCs	SCX	Alberton et al. (2012)
Nonviral vectors	Perichondrial cells	TGF-β	Goomer et al. (2000)
Nonviral vectors	Tendon cells	BMP-7	Groth et al. (2017)

Abbreviations: rAAV, recombinant adeno-associated virus; MSCs, Mesenchymal stem cells; IGF-1, insulin-like growth factor-1; BMP, bone morphogenetic proteins; TGF-β, transforming growth factor-β; VEGF, vascular endothelial growth factor; SCX, scleraxis; MKX, mohawk; FGF, fibroblast growth factor.

##### 5.4.2.2 Transcription factors

Transcription factors have shown great promise and unique advantages in repairing ACL injuries (Liu et al., 2014). In particular, scleraxis (Scx), Mohawk (Mkx), and early growth response factor (Egr1) have demonstrated key roles in ACL development, maturation, homeostatic maintenance, and repair (Asahara et al., 2017; Gulotta et al., 2011). SCX is mainly responsible for regulating cellular differentiation and matrix synthesis, whereas MKX affects ACL differentiation by regulating mechanical property components. Egr1 is expressed during ACL development and has important roles in ligament formation (Asahara et al., 2017; Liu et al., 2014; Otabe et al., 2015; Alberton et al., 2012). These findings emphasized the importance of transcription factors in ACL injury repair, suggesting they are key molecules in promoting recovery in damaged ligaments. By gaining a deeper understanding of their specific roles and regulatory mechanisms, new transcription factor-based therapeutic strategies may be developed to effectively promote ACL injury repair and regeneration (Table 3).

##### 5.4.2.3 Anti-inflammatory agents

In early ACL healing stages, the anti-inflammatory IL-1 receptor antagonist (IL-1Ra) promoted ACL healing by stimulating M2-type macrophages and altering granulation tissue composition, which created a local, low inflammatory response environment (Chamberlain et al., 2013). Soluble tumor necrosis factor-α (TNF-α) receptors also repaired damaged ligaments, inhibited local inflammatory responses, and prevented complications such as osteoarthritis, which may occur after ACL injury (Lawrence et al., 2011). Additionally, the regulatory cytokine IL-10 acted as a positive antagonist of TNF-α in local knee joint environments after ACL injury, thus helping to prevent osteoarthritis after ACL injury (Bigoni et al., 2019). These findings highlighted the critical role of anti-inflammatory factors in treating ACL injuries, especially when controlling inflammatory responses and promoting healing processes. Such anti-inflammatory factors may significantly improve recovery processes after ACL injury by effectively modulating local inflammatory responses in knee joints, thereby reducing complication risks. By exploiting these factors, researchers can better understand complex biological responses following ACL injury, thereby improving therapeutic outcomes and accelerating recovery. Such advances are not only important for treating ACL injuries, but provide possible treatments for other ligament and soft tissue injuries.

##### 5.4.2.4 Matrix components

ACL contains collagen fibers, tenomodulin, core proteoglycans, fiber regulatory proteins, and disaccharide chain proteoglycans, which help maintain natural ACL structure and functional integrity. Therefore, repairing injured ACLs by transfecting vectors expressing matrix component genes may be a worthwhile therapeutic approach to naturally replicate original biological and mechanical properties in tissues (Page et al., 1992; Evangelopoulos et al., 2017; Lewis et al., 2010; Dunkman et al., 2014; Delalande et al., 2015).

#### 5.4.3 Gene therapy limitations

Although gene therapy is a promising approach, inherent issues persist that require consideration. Firstly, due to differences in transfection methods, transfected gene expression may decrease or be lost over time. This means that gene transfection strategies must be continuously optimized and adjusted to achieve long-lasting and effective therapeutic effects. Secondly, gene therapy technologies face challenges with respect to biosafety. During treatment processes, there may be a risk of gene mutation, which can lead to unexpected biological effects or adverse reactions. Therefore, it is crucial to ensure method safety, which requires strict monitoring and the management of possible gene mutations during treatments. Finally, identifying ideal vectors is an important research area. Such vectors should have high efficiency, stability, and safety traits in ensuring gene therapy effectiveness and patient safety. These issues must be explored and improved through continuous research and trials. Therefore, gene therapy has great potential, but several technical and safety challenges must be overcome. Through future research and innovation, gene therapy will have important roles in many diverse medical fields.

### 5.5 Self-assembled short peptide (SAP) therapy

With rapid developments in biotherapeutic, regenerative medicine, and tissue engineering fields, SAPs have attracted much attention as emerging nanomaterials in tissue engineering. SAPs are mainly composed of amino acids, which mimic extracellular matrix properties (Luo and Zhang, 2012), and have major potential in tissue engineering and regenerative medicine fields. As biomimetic materials, SAPs provide environments similar to the natural extracellular matrix in supporting cell attachment, growth, and differentiation, traits critical in tissue repair and regeneration. Additionally, due to nanoscale and controlled biocompatibility properties, SAPs offer vast possibilities in designing and developing novel tissue engineering materials. By comprehensively examining SAP properties, their use in healing and functional recovery of injured tissues is promising.

SAP as a biological tissue engineering technology concept, although it emerged in 2022, but because it is still in an emerging stage, related research has not been widely carried out, resulting in the current published literature around this topic is rather sparse. In view of the initial state of this scientific research activity, it is obviously not appropriate to conduct accurate statistical analysis of SAP academic output and predict the trend of future publications, so the author has not carried out specific bibliometric work on this at the current stage.

#### 5.5.1 SAP therapeutic advantages

As innovative tissue engineering materials, SAPs have significant advantages: good biocompatibility, no immune rejection issues, no cytotoxicity, and they form three-dimensional scaffold structures similar to the extracellular matrix. These properties make SAPs highly promising materials in the biomedical field, especially in tissue engineering and regenerative medicine. Studies have reported that one of the potential mechanical reasons for self-healing failure after ACL injury is the premature loss of trabecular temporary scaffolds (Tovar et al., 2012). SAPs can provide mechanical support for neoplastic cells via scaffold-like structures, which may become novel repair strategies for ACL injuries. Animal studies have shown that SAPs promote ACL injury repair in rabbits to some extent. SAPs have been demonstrated to effectively stimulate not only the functional activity and proliferative capacity of ACL fibroblasts but also the expression of both COL1 and collagen type III, thereby indicating their multifaceted role in modulating ACL tissue homeostasis. Addition, SAPs may be used as three-dimensional culture substrates for ACL fibroblasts, which adhere to and control growth factor release, thus promoting ACL injury repair. Therefore, SAPs provide new possibilities for treating ACL injuries, and are expected to improve repair and recovery processes after injury. With further research on associated properties and applications, SAPs have an exciting prospect for treating ACL injuries.

#### 5.5.2 SAP therapy limitations

Using SAPs to repair damaged ACLs allows for minimal invasive manipulation and promising clinical applications. SAPs provide new and potentially non-reconstructive treatment modalities for ACL injury repair. However, more clarification is required regarding specific SAP molecular mechanisms in ACL repair. Understanding these processes is crucial for optimizing SAP applications, along with improving therapeutic efficacy and ensuring treatment safety. Overall, SAPs demonstrate innovative and potential non-surgical treatment avenues for ACL injuries. With future research, SAPs may provide more diverse therapeutic options for patients with ACL injuries.

### 5.6 Platelet-rich plasma (PRP) therapy

PRP preparations are generated by centrifuging the patient’s blood and extracting fractions rich in active platelets. These fractions are then be injected into damaged ACL joint fluid. Platelets generate many growth factors, including FGF, TGF-β, IGF, PDGF, and VEGF. PRP preparations may be directly injected into damaged areas or combined with other cells/proteins to generate gel scaffolds before implantation. The latter approach is more effective in promoting ligament fibroblast proliferation and collagen production, and is also more stable in grafts. As a result, PRP therapy significantly enhances tissue healing and angiogenesis (Moraes et al., 2013; Bava and Barber, 2011). Also, using the patient’s own blood, PRP provides an effective biotherapeutic strategy for treating ACL injuries, and shows great potential in improving healing efficiency and optimizing recovery processes. Thus, PRP therapy may become more clinically significant in treating ACL injuries.

### 5.7 BMAC

Bone marrow aspirate concentrate (BMAC) is a component rich in bone marrow mononuclear cells, isolated from the bone marrow through centrifugation, containing stem cells ready for use without the need for *ex vivo* expansion. BMAC can be harvested from the patient’s iliac crest during surgery or collected from the proximal region of the femur, and its widespread availability makes it ideal for clinical settings. In addition to mesenchymal stem cells, a successful BMAC harvest also contains hematopoietic stem cells, platelets rich in growth factors, BMPs, and other progenitor cells (Hogan et al., 2021). BMAC is believed to enhance the treatment of tendon injuries, knee osteoarthritis, and intervertebral disc degeneration, as well as allograft tendon regeneration and tendon-bone tunnel interface healing. The stem cells and growth factors contained in BMAC can promote the proliferation and differentiation of tendon cells, accelerating the healing process of tendon injuries (Aydın et al., 2023). A retrospective, single-center study showed that BMAC-enhanced open repair of sports-related Achilles tendon ruptures led to 92% of patients resuming sports without re-rupture (Stein et al., 2015). Study results indicate that the combination of BMAC and PRP demonstrates enhanced clinical outcomes, graft maturation, and tendon-bone interface healing compared to traditional ACL reconstruction surgery with PRP alone or without other biological enhancements (Lin et al., 2024). MSCs contained in BMAC can create a microenvironment that promotes tissue regeneration, stimulates angiogenesis, and reduces the formation of scar tissue. MSCs can also differentiate into various cell lines, including bone, cartilage, and ligament tissues, and recruit cells to facilitate the healing process (Forsythe et al., 2022). BMAC therapy is an emerging biological repair method for ACL injury that has emerged in recent years. Although BMAC has shown positive results in some preliminary studies, there is still a lack of large-scale, high-quality clinical trials to support its long-term efficacy and safety. Currently, the application of BMAC lacks uniform treatment standards, including optimal dosage, injection frequency, and duration of treatment. The effectiveness of BMAC may be influenced by patient-specific factors, such as age, health status, and type of injury, which means that BMAC may not be suitable for all patients. Since BMAC is extracted from the patient’s own bone marrow, there may be significant variations in BMAC components between different patients, which could affect the consistency of treatment outcomes. Although BMAC has shown promise in some clinical applications, these limitations need to be addressed, and more research is required to enhance the evidence base for its scientific foundation and clinical effectiveness (Hogan et al., 2021).

### 5.8 Extracorporeal shock wave therapy

Extracorporeal shock wave therapy (ESWT) is an advanced non-invasive medical technique that utilizes high-energy shock waves to promote tissue repair and pain alleviation. Originating from urological lithotripsy, ESWT has been successfully extended to various medical fields, including the musculoskeletal system. During the treatment, the mechanical pressure generated by the propagation of shock waves within the tissue activates cellular activity, accelerating tissue repair. Concurrently, these waves can stimulate cellular signaling pathways, promoting the release of growth factors that further aid in tissue healing. Additionally, the temporary local vasodilation induced by ESWT increases blood flow, improving local circulation and oxygen supply, thereby delivering the nutrients required for the regeneration of damaged tissue. The analgesic effects of ESWT are partly attributed to its stimulation of pain-inhibiting pathways, particularly the release of endorphins (Takase et al., 2024). A study on the efficacy of Radial Extracorporeal Shock Wave Therapy (rESWT) in patients who underwent ACLR showed that rESWT is an effective and positive rehabilitation method during the early recovery period of ACLR, reducing patients’ pain levels and improving knee joint function (Song et al., 2024). The results of a study by Weninger et al. (2023) indicated that ESWT can significantly shorten the time to return to rotational sports and running activities, and increase the number of patients reaching pre-injury activity levels, making it a cost-effective treatment option with minimal side effects after ACL reconstruction. Although ESWT is widely used in the repair following anterior cruciate ligament injury, high-quality clinical evidence regarding its effectiveness remains limited, with many studies being small-scale and lacking long-term follow-up. Determining the optimal number of treatment sessions and duration of treatment remains a challenge, as overtreatment may not offer additional benefits and could potentially increase the risk of side effects (Rahim et al., 2022). To achieve broader application in medical practice, these limitations need to be addressed, and further research is required to enhance the scientific foundation and evidence base for its clinical effectiveness.

### 5.9 Electrical stimulation therapy

Electrical stimulation therapy, as an innovative medical approach, has demonstrated unique advantages in the treatment of musculoskeletal disorders, particularly ligament injuries. This method is not only cost-effective and safe but also highly convenient to use. As a minimally invasive or non-invasive treatment modality, it aligns well with the treatment principles advocated in the field of orthopedic sports medicine today. In the treatment of ACL injuries, electrical stimulation therapy, especially neuromuscular electrical stimulation (NMES), has proven its significant value in restoring motor function. NMES, by stimulating muscle contractions, not only promotes the healing of damaged muscles but also plays an active role in muscle rehabilitation after ACL reconstruction. This therapy can significantly improve muscle strength, increase range of motion, reduce edema, slow down muscle atrophy, accelerate tissue healing, and effectively alleviate pain, even having a positive impact on muscle enzyme activity (Arhos et al., 2024; Ilfeld et al., 2021).

In a randomized controlled trial examining the use of NMES after ACL injury and reconstruction, NMES significantly reduced muscle fiber atrophy, particularly affecting the fast myosin heavy chain (MHC II) fibers. At the same time, NMES also maintained the contractile ability of slow myosin heavy chain (MHC I) fibers, increased the maximum contraction speed, and maintained power output, despite having a minimal impact on MHC II fibers (Toth et al., 2020). Furthermore, NMES has shown its utility in alleviating pain, knee joint swelling in patients after ACL reconstruction, and reducing the incidence of deep vein thrombosis (DVT), becoming a simple and effective interventional treatment method (Xiong et al., 2022). After ACL reconstruction, effective analgesia is not only crucial for pain control but also helps to reduce the use of opioid medications and promotes early recovery. Given the considerable number of opioid prescriptions issued by orthopedic physicians, the potential of NMES to reduce the consumption of these drugs is particularly prominent (Hurley et al., 2023).

However, we must also recognize that the subjectivity of pain perception means that the effectiveness of electrical stimulation treatment largely depends on the patient’s self-report, which can be influenced by multiple psychological and social factors. Although electrical stimulation treatment has been widely used in pain management for ACL injuries, the exact mechanisms of its action are not fully understood, which to some extent limits the optimization and personalized adjustment of treatment. Therefore, to gain a deeper understanding of the potential of NMES and to apply it more broadly in the treatment of ACL injuries, there is an urgent need for more high-quality research to provide a solid scientific basis and guidance.

### 5.10 Cross bracing protocol

The prevailing view holds that once the ACL is ruptured, its capacity for self-healing is limited. However, anatomical studies have shown that the ACL has a rich blood supply, and histological studies have described that the ACL undergoes typical healing phases after injury, albeit at a slower rate and with reduced healing capacity compared to the medial collateral ligament. Cross bracing protocol (CBP), a non-surgical treatment method for ACL ruptures. Its primary goal is to attempt to promote the healing of the ACL. The specific methods include:

1. In the acute phase after ACL rupture, immobilize the knee joint in a brace at 90° flexion for 4 weeks.
2. After 4 weeks, gradually increase the range of motion of the knee joint, with adjustments made weekly.
3. Remove the brace at 12 weeks.
4. Combine with rehabilitation exercises supervised by a physiotherapist, which include target-oriented lower extremity neuromuscular control, muscle strengthening, and power training, as well as functional training aimed at helping patients resume sports and leisure activities.

A key feature of the CBP is the initial use of a brace to immobilize the knee joint at 90° flexion, based on anatomical studies that found the shortest distance between the origin and insertion points of the ACL at this angle, which may help reduce the gap between the torn ligament stumps, promoting tissue bridging and healing. The CBP also includes standardized, target-oriented, movement-based rehabilitation exercises under the guidance of a physiotherapist, as well as continued rehabilitation exercises during and after the use of the brace. Some studies have shown that after treating acute ACL ruptures with the CBP, 90% of patients had evidence of ACL healing on MRI at 3 months, and greater ACL healing on the 3-month MRI was associated with better outcomes. However, there is a scarcity of research on promoting the healing of ACL injuries with the CBP method, and longer-term follow-ups and clinical trials are needed to guide clinical practice (Filbay et al., 2023).

## 6 Conclusion

**FIGURE 5:**
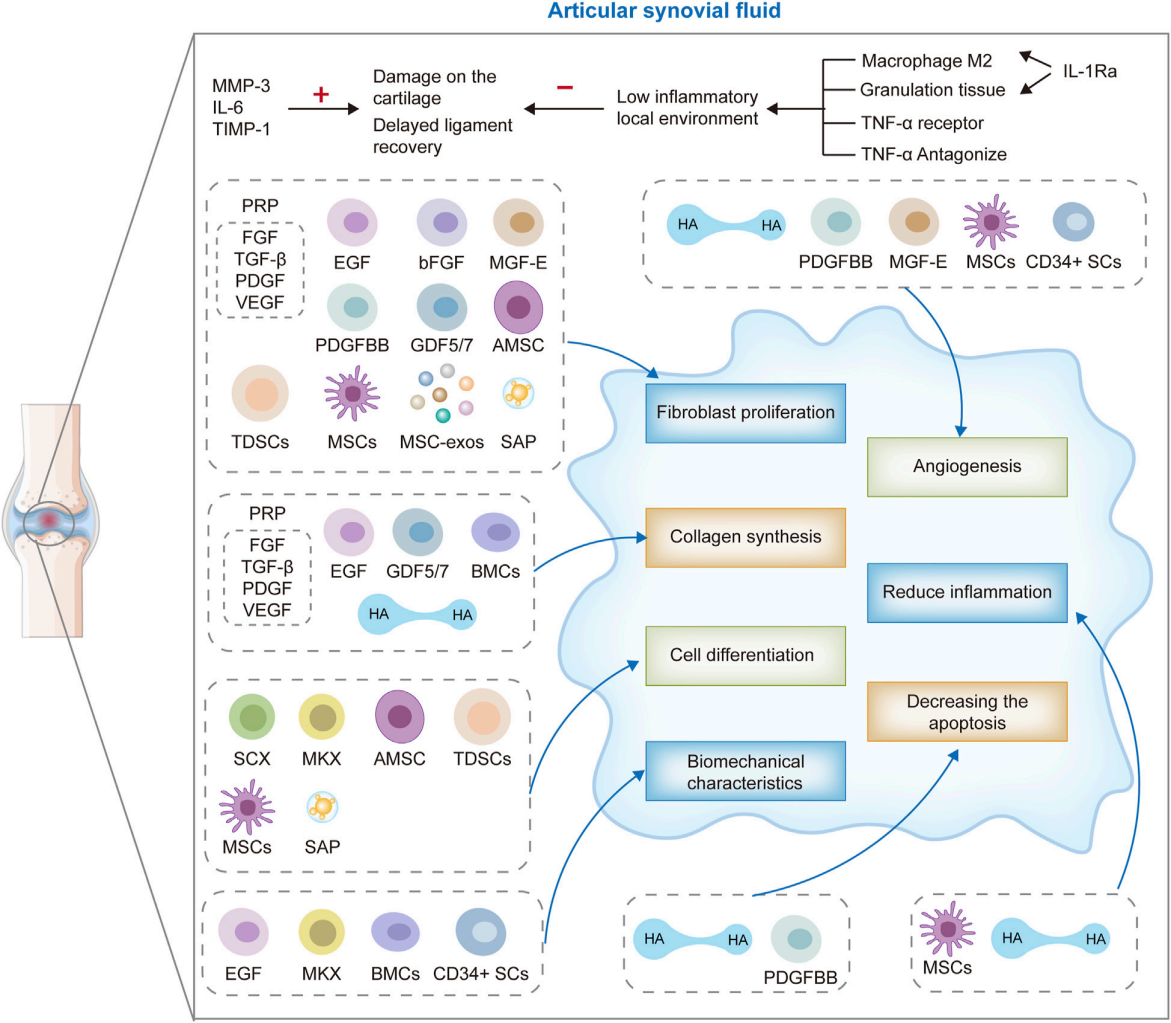
Effects of joint fluid, stem cells and growth factors on ACL repair.

ACL serves as a crucial stabilizer for knee integrity, yet ACL injuries present a formidable challenge to athletes and the general population alike. These injuries can result in chronic functional limitations and a heightened risk of osteoarthritis. While ACL reconstruction has been the cornerstone of traditional treatment, the quest for more efficacious and minimally invasive alternatives has catalyzed a surge in interest surrounding biological therapies. This paper reviews the recent advances in biological therapy of ACL injury, including HA, SAP, growth factor, stem cell, gene therapy and PRP, BMAC, ESWT, electrical stimulation and CBP (Figure 5). With the continuous progress of bioengineering, the technology combining hyaluronic acid (HA), stem cells, growth factors, and gene expression vector and Scaffold has become a new trend in the treatment of anterior cruciate ligament (ACL) injury. This multi-strategy approach is designed to simulate the natural microenvironment of ACL and facilitate the repair and regeneration of damaged ligaments by providing the necessary bioactive molecules and mechanical support. The viscoelastic properties of HA help to protect and lubricate cells, while stem cells provide a new source of cells for the site of injury. Growth factors can promote cell proliferation and differentiation, gene expression vectors can deliver therapeutic genes directly to target cells, thereby activating the self-repair mechanism of ACL, ESWT and electrical stimulation reduce pain, and CBP can help better bridge damaged ligaments and facilitate functional recovery. The combined use of these technologies in the future will not only improve the treatment effect, but also provide new possibilities for personalized medicine and precision therapy. Although gene therapy is still in its initial stage, with the development of sequencing technology, it has great prospects in precisely controlling the expression of therapeutic genes to drive ACL healing through whole transcriptome sequencing and single cell sequencing, etc. Despite these encouraging findings, some challenges and limitations remain. To achieve widespread adoption and successful implementation of biologic therapies in clinical practice, issues that must be addressed include the variable and sometimes transient nature of treatment effects, the need to standardize preparation methods and dosing regimens, the likelihood of immune reactions or adverse events, and the high costs associated with some biologic therapies.

Looking ahead, future research should focus on:

1) Efficacy optimization: Conduct well-designed controlled clinical trials with larger sample sizes and longer follow-up periods to determine the true efficacy of these biologic therapies, especially compared to traditional ACL reconstruction methods.
2) Elucidation of mechanisms: Further study of the molecular and cellular mechanisms of therapeutic action of these biologics will contribute to the development of more targeted and effective therapies.
3) Combination therapy: Explore the synergies of different biological therapies combined or combined with conventional surgical techniques to achieve better healing results.
4) Improve drug delivery systems: Develop advanced drug delivery systems, such as biomaterial-based scaffolds or encapsulation technologies, to ensure controlled release and targeted delivery of biologics to the injured ACL site.
5) Personalized medicine: Identify patient-specific factors that predict treatment response, allowing the application of customized biologic therapies to maximize outcomes for individual patients.

In conclusion, the emergence of biologic therapies offers a promising avenue to revolutionize ACL injury management, with the potential to enhance healing, reduce postoperative complications, and improve long-term functional outcomes. Continued research and technological advances will help overcome current limitations, refine treatment options, and ultimately translate these innovative therapies into clinical practice, ushering in a new era of personalized, biologically driven ACL repair strategies.
